# Growth, Quantitative Growth Analysis, and Applications of Graphene on γ-Al_2_O_3_ catalysts

**DOI:** 10.1038/srep11839

**Published:** 2015-07-03

**Authors:** Jaehyun Park, Joohwi Lee, Jung-Hae Choi, Do Kyung Hwang, Yong-Won Song

**Affiliations:** 1 Center for Opto-Electronic Materials and Devices, Korea Institute of Science and Technology, Hwarangno 14-gil 5, Seongbuk-gu, Seoul 136-791, Republic of Korea; 2 Center for Electronics Materials, Korea Institute of Science and Technology, Hwarangno 14-gil 5, Seongbuk-gu, Seoul 136-791, Republic of Korea

## Abstract

The possibilities offered by catalytic γ-Al_2_O_3_ substrates are explored, and the mechanism governing graphene formation thereon is elucidated using both numerical simulations and experiments. The growth scheme offers metal-free synthesis at low temperature, grain-size customization, large-area uniformity of electrical properties, single-step preparation of graphene/dielectric structures, and readily detachable graphene. We quantify based on thermodynamic principles the activation energies associated with graphene nucleation/growth on γ-Al_2_O_3_, verifying the low physical and chemical barriers. Importantly, we derive a universal equation governing the adsorption-based synthesis of graphene over a wide range of temperatures in both catalytic and spontaneous growth regimes. Experimental results support the equation, highlighting the catalytic function of γ-Al_2_O_3_ at low temperatures. The synthesized graphene is manually incorporated as a ‘graphene sticker’ into an ultrafast mode-locked laser.

Owing to its excellent electrical and optical characteristics, graphene has attracted much research attention over the past decade^1^,^2^,^3^. Since the demonstration of mechanical exfoliation to prepare graphene in 2004^1^, various processes have been developed to scale-up the production of two-dimensional crystals. These include drop-casting, wide-area spraying and vacuum filtration of graphene oxide flakes^4^, the epitaxial synthesis of graphene from wafer-scale SiC via sublimation at high temperatures^5^, and the roll-based transfer of graphene from Cu foil via chemical vapor deposition (CVD)^6^. However, these processes have not been able to match mechanical exfoliation in terms of the quality of grapheme produced.

Transfer-free processes have drawn particular interest because the transfer of large graphene sample synthesized on a metal catalyst generally leads to contamination or the formation of defects^7^. Moreover, transfer-free methods are applicable to non-planar curvilinear or irregular surfaces. Direct growth schemes on metal oxide substrates are promising in this context because apart from avoiding deleterious transfers, the metal oxide substrates act as insulating components as well. Catalytic graphene growth has been explored on sapphire^8^,^9^,^10^,^11^, quartz^12^,^13^, and SiO_2_^13^ with the spontaneous dehydrogenation of C precursors; however, these are all high-temperature reactions. Similarly, while graphene layers have been grown at the interface of a Ni film and an insulating substrate at relatively low temperatures^14^,^15^,^16^, this transfer-free approach still requires thermal pretreatment of the Ni films at more than 800 °C, leading to the thermal diffusion of Ni into the insulating substrates and the formation of ternary phases, which in turn causes malfunctions in the resulting electronic devices^17^,^18^,^19^. (Note that Ni diffusion occurs at 300 K on SiO_2_ substrate.) Recently, the use of catalytic MgO^20^,^21^ or ZrO_2_^21^ substrates has been shown to dramatically lower the processing temperature required. However, the graphene layers produced were only submitted to a very limited analysis and their poor quality and the small crystals therein clearly limit their application in practice. A new insulating catalytic substrate is therefore required that guarantees low-temperature and transfer-free growth of high-quality graphene. Moreover, a quantitative analysis of the energy factors involved in the formation of crystals is desirable to precisely control their size, morphology, and quality.

In this report, we explore γ-Al_2_O_3_ catalyst as a novel substrate for the metal-free direct growth of graphene layers at low temperatures. We elucidate the formation mechanism of graphene on γ-Al_2_O_3_ with both numerical simulations and experiments, with concordant results obtained. We derive an equation applicable universally for the adsorption-based synthesis of graphene over a wide range of temperatures, which covers both low-temperature catalytic growth and high-temperature spontaneous growth on diverse substrates, including metal catalysts. We show herein that this equation accurately reproduces experimental results. To verify the successful formation of graphene crystals^2^,^3^, we demonstrate an ultrafast mode-locked laser incorporating one of our readily detachable graphene layers, which was transferred manually to the nonlinear photonic device as a ‘graphene sticker’.

## Results

Graphene synthesized on γ-Al_2_O_3_ substrates is advantageous in a number of regards over conventionally prepared graphene; these include (i) the low-temperature synthesis afforded by γ-Al_2_O_3_ catalysis, (ii) the excellent and very uniform electrical properties provided by the ultrahigh-quality crystals that form because of the negligible activation energies associated with carbon diffusion on the substrate and with the surface-regulated crystal nucleation and growth, (iii) the control offered over the thickness of the graphene layer and of the grain size therein—from nano- to micrograins, which is closely related to band gap opening, (iv) the fact that the formation mechanism is demonstrated here both numerically and experimentally, and (v) the ready detachability of the graphene produced and its very low adsorption energy with respect to the substrate, which guarantee the complete transfer of intact layers. Furthermore, the graphene grown can be used with the γ-Al_2_O_3_ substrate—an ideal gate dielectric with a high dielectric constant (κ) of 7.9^22^—in graphene transistors^23^, with a low leakage current density afforded by the large band gap (~8.2 eV, in spite of its crystalline nature^24^) of the substrate. In contrast, although the high catalytic potential of MgO^20^,^21^ and ZrO_2_^21^ has been shown to allow graphene growth at temperatures as low as ~350 °C, the high surface diffusion barrier for C adatoms on these crystals and their small band gaps respectively lead to uneven graphene growth^20^,^21^ and to high leakage current densities (~1 × 10^−4^A·cm^−2^)^25^,^26^.

**Figure 1:**
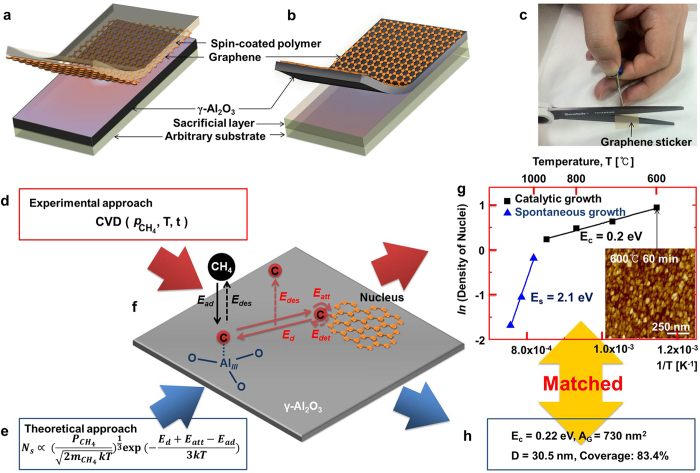
Schematic illustration of the preparation of readily detachable graphene on γ-Al_2_O_3_ substrates, which can be used directly after mechanical detachment. **a**, Graphene covered with a spin-coated polymer film and **b**, with a γ-Al_2_O_3_ substrate formed on a sacrificial layer. **c**, Photograph of a graphene sticker (graphene with a spin-coated polymer film), which is easily tailored using scissors. **d**, The chemical vapor deposition parameters used for these experiments. **e**, The theoretically derived growth equation governing the saturation density of nuclei (N_s_). P_CH4_, T, and t respectively indicate the partial pressure of CH_4_, and the growth temperature and time, while m_CH4_ and k stand for the mass of CH_4_ and the Boltzmann constant, respectively. **f**, Schematic illustration of the nucleation and growth of graphene on γ-Al_2_O_3_. The Al_*III*_ site is the catalytically active site for the adsorption and dehydrogenation of CH_4_. The bold solid and dotted lines respectively indicate the supply and withdrawal of the C adatom during graphene formation. **g**, Natural logarithm of the density of graphene nuclei as a function of inverse temperature. This plot was used to estimate the activation barriers associated with the growth of graphene on γ-Al_2_O_3_. The inset atomic force micrograph is taken from a graphene sample grown at 600 °C for 1 h, and corresponds to the point at 600 °C in the Arrhenius plot. **h**, The activation barrier (E_c_), the average grain area (A_G_), and the grain diameter (D) and total coverage of graphene calculated theoretically for growth at 600 °C for 1 h. The corresponding experimental results are shown in the atomic force micrograph in (g).

Figure 1a shows the schematic illustration of the fabrication of the graphene sticker, which consists of a polymer spin-coated onto the grown graphene detached by peeling-off the polymer film^22^. Figure 1b illustrates the exfoliation of graphene/γ-Al_2_O_3_ from the additional sacrificial layer deposited on the arbitrary substrate to form a graphene/high-κ dielectric. The graphene can be submitted to additional treatments with the assistance of supporting substructures to obtain ultrathin, transparent sheets and a highly bendable Al_2_O_3_ substrate. In particular, graphene stickers, as shown in Fig. 1c, can be prepared that can simply be cut out with scissors for applications in flexible or stretchable electronic or photonic devices.

To evaluate the versatility of γ-Al_2_O_3_ as a substrate for growing graphene, we analyzed the growth mechanism theoretically and validated the resulting equation experimentally. An initially amorphous Al_2_O_3_ layer was prepared by atomic layer deposition (ALD). This spontaneously transforms into γ-phase during the first stage of graphene growth, and this phase is retained as the substrate is recycled after growing and detaching the graphene. (The specific conditions used for these steps are described in the methods section)^22^.

The γ-Al_2_O_3_ substrate formed was used for the growth of graphene using CVD at different CH_4_ partial pressures (p_CH4_), growth temperatures (T), and durations (t), as shown in Fig. 1d. The growth related equation shown in Fig. 1e was derived as described below and accurately reproduces the experimental results.

To confirm the catalytic activity of the γ-Al_2_O_3_ substrate and the growth characteristics of graphene, graphene layers were grown at temperatures ranging from 600 °C to 1050 °C. Regardless of the activity of the substrate, graphene always grows under CVD conditions at temperatures above 950 °C via the spontaneous dehydrogenation of CH_4_^27^. Below 950 °C however, graphene can only grow at catalytic sites on the surface of the substrate. The catalytic activity of γ-Al_2_O_3_ can therefore be proved experimentally by demonstrating graphene growth at temperatures below 950 °C. According to previous reports^28^,^29^, CH_4_ is only adsorbed at tricoordinated Al_*III*_ sites on the surface of γ-Al_2_O_3_. Furthermore, these sites show catalytic activity for dehydrogenation at temperatures as low as 100 °C^28^,^29^. Figure 1f illustrates the formalism we have developed to describe graphene growth on γ-Al_2_O_3_ substrates. This scheme contains a number of activation barriers with many factors, which are described below.

A carbon adatom can be generated at Al_*III*_ sites by dissociative adsorption of a CH_4_ molecule. The overall activation energy barrier from the CH_4_ molecule to the generation of a C adatom involves the dissociative adsorption energy, E_ad_. The generated adatom then diffuses and becomes anchored onto sites that are more or less favorable according to their associated surface diffusion barrier, E_d_. Additional C adatoms may come into contact with the anchored C adatom to form linear and ring shaped carbon clusters, as described by the attachment barrier, E_att_. Alternatively, the C adatom may desorb from the γ-Al_2_O_3_ substrate immediately after its generation or as it diffuses across the surface according to the desorption energy, E_des_. The activation energy for the detachment of a C atom from the perimeter of the graphene nucleus is the detachment barrier, E_det_.

After growing graphene at various temperatures, we measured the overall activation barrier for growth (E_a_) via Arrhenius plots, as shown in Fig. 1g. This E_a_ includes all contributions from E_ad_, E_d_, E_att_, and E_des_, and the complex relationship between these factors. Measuring E_a_ provides an experimental parameter against which to validate our theoretical model, and provides insights into the formation and growth of the graphene nuclei.

### Experimental measurement of overall activation barriers

To sketch out the overall growth of graphene nuclei on the γ-Al_2_O_3_ substrates, we extracted E_a_ by modeling the experimental data using the Arrhenius equation. Plotting the natural logarithm of the density of graphene nuclei against the inverse of the growth temperature (in Kelvin) reveals two distinct growth regimes. The gentle slope in Fig. 1g, corresponding to an energy barrier E_c_ = 0.21 eV, represents E_a_ in the catalytic growth regime, while the steeper slope reflects the non-catalytic regime with E_s_ = 2.10 eV, in which growth occurs through site independent spontaneous dehydrogenation of CH_4_. The specific procedure used to obtain these values is described in Figure S1 in the Supplementary Information (SI).

Based on these theoretical considerations, E_c_ and E_s_ should be given by Equations (1) and (2), respectively.


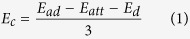



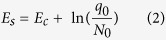


In the above equation, E_ad_ = 0.88 eV, E_att_ = 0.21 eV, E_d_ = ~0 eV, and ln(q_0_/N_0_) = 1.87 eV, for which N_0_ = 26 / 0.76 nm^−2^ and q_0_ = 4 / 0.76 nm^−2^ represent the total density of adsorption sites respectively on the surface of the substrate and for CH_4_ on γ-Al_2_O_3_; i.e., there are 26 atoms in total and 4 Al_*III*_ atoms on each γ-Al_2_O_3_ unit cell (with an area of 0.76 nm^2^)^30^.

Inserting these values into the above equations leads to E_c_ ~ 0.22 eV and E_s_ ~ 2.09 eV, as shown in Fig. 1h, in good agreement with the experimental behavior (E_c_ = 0.21 eV and E_s_ = 2.10 eV) shown in Fig. 1g.

### Theoretical derivation of the growth mechanism

Ruoff and coworkers have shown that graphene synthesis on transition metals involves surface adsorption on Cu but surface segregation or precipitation on Ni due to the differing solubility of C in the two (viz. a negligible <0.001 at% in Cu, but ~1.3 at% in Ni at 1000 °C)^31^,^32^. For Al_2_O_3_, Al_4_C_3_ forms very slowly and only above 1600 °C, while Al_2_OC, which is the first metastable product in the formation of Al_4_C_3_, forms only at temperatures above 1427 °C^33^. The solubility of C in bulk Al_2_O_3_ is therefore negligible under the CVD conditions employed here, such that the formation and growth of graphene nuclei on γ-Al_2_O_3_ should follow a surface adsorption route similar to that which occurs on Cu. Two-dimensional crystal growth is similar to the nucleation and enlargement of crystals synthesized by physical deposition. We therefore adapted the model for the latter, derived by Robinson and Robins^34^, to the growth mechanism investigated here, leading to the following universal equation governing the adsorption-based synthesis of graphene over a wide temperature range. (The specifics of this derivation appear on page S3 of the SI.)


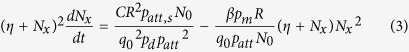


In the above equation, η is used as a determinant for the growth regime, β is an undetermined dimensionless coefficient, C is the number of effective pair-formation sites neighboring each adatom, and the other factors are described in the SI. The determinant is highly dependent on the validity of the values used for the two activation barriers, E_des_ and E_d_, at a given growth temperature. The saturation densities in the high-temperature and catalytic growth regimes are derived from the Equation (3), as shown in Equations (4) and (5), respectively.


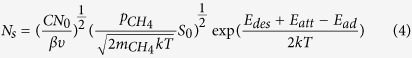



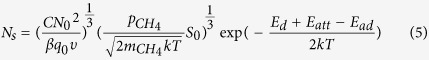


However, the value calculated for E_a_ in the high temperature growth regime does not match the one measured experimentally. We therefore obtained a rough estimate of the transition temperature (T_c_) from the intersection of the lines corresponding to Equations (4) and (5), yielding T_c_ ≈ 4800 K (page S13 and Figure S3 in the SI). Hence, under the CVD conditions used here, graphene growth should be governed by Equation (5), and another approach is necessary to account for the catalytic growth evidenced in Fig. 1g, which should in turn be divided into two regimes. The solution lies in the fact that the number of CH_4_ adsorption sites varies with the growth temperature (see page S15 in the SI).

The redefined adsorption rate (R′) is given by the following equation,


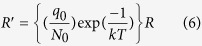


and the activation barriers in the steeper regime governed by spontaneous dehydrogenation (E_s_) are defined as follows:


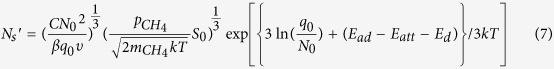


with the values for E_a_ in the catalytic and spontaneous growth regimes given by Equations (1) and (2), respectively.

### The initial nucleation rate at all temperatures

From Equation (3), the initial nucleation rate, J, can be predicted from the value of dN_x_/dt at t = 0 and N_x_ = 0.


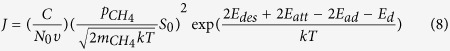


Equations (4), (5), (7) and (8) constitute to the best of our knowledge, the first description of the growth mechanisms of nanocarbon (either graphene, carbon nanotubes, or fullerene) that covers the entire process from the decomposition and adsorption of the C precursor to the nucleation and growth of nanocarbon.

**Figure 2:**
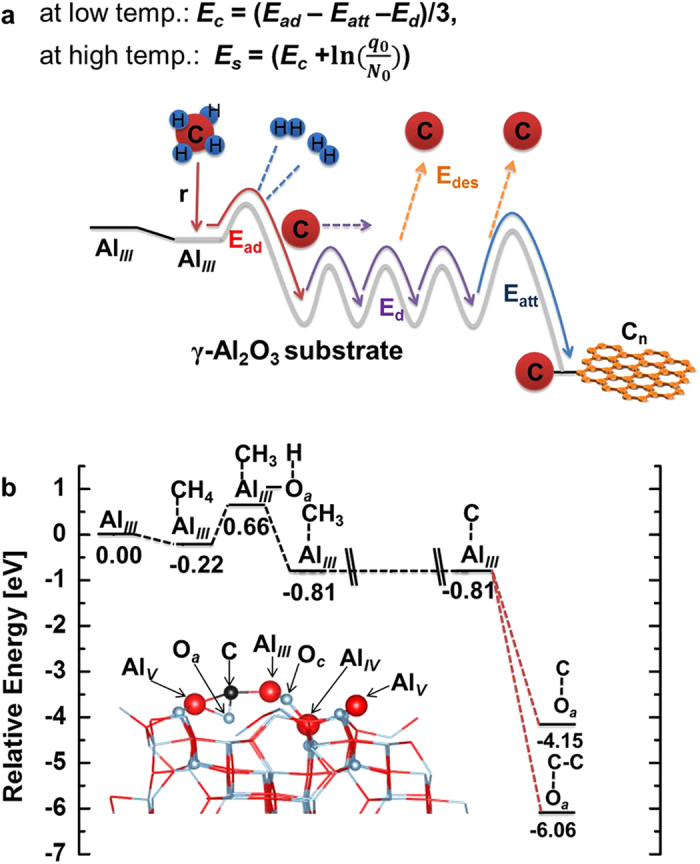
Energy path for the formation of graphene nuclei. **a**, The energy landscape for the sequential formation of graphene nuclei on a γ-Al_2_O_3_ substrate. E_c_ and E_s_ represent the overall activation barriers for nucleus growth at different temperatures. **b**, Minimum energy path for the formation of graphene nuclei. The inset shows the result of the most stable state of a C adatom on the surface of a γ-Al_2_O_3_ substrate.

Figure 2a presents a schematic illustration of the energy landscape, based on Fig. 1f, associated with graphene growth, from CH_4_ dissociation to the formation of graphene nuclei on the γ-Al_2_O_3_ substrates. Figure 2b shows the results of density functional theory (DFT) calculations performed for a C adatom based on reference data for the dissociative adsorption of CH_4_^28^,^29^. Whole atoms—including O sites adjacent and non-adjacent to the initial Al_*III*_ site, and other Al sites—were considered as potential destinations for the surface diffusion of an adatom generated from the dissociative adsorption of CH_4_ on an Al_*III*_ site. These calculations indicate that these other Al sites are more unstable destinations than the original Al_*III*_, and that O sites both adjacent and non-adjacent to the original Al_*III*_ site, are more stable than the latter. Examples of the DFT calculations performed for these O sites are provided in Figures S5–7 in the SI. These indicate that the O_*a*_ site adjacent to the initial Al_*III*_ site is the most stable (see Figure S5c,f).

### Evaluation of the compositional activation barriers

Figure 2b shows that E_ad_ in this situation is equal to 0.88 eV. Such a low energy barrier ensures the dissociative adsorption of CH_4_ to C adatom, which is the rate-determining step^35^,^36^ in the growth mechanism, sets the lower limit of the growth temperature at 100 °C^28^,^29^.

**Figure 3:**
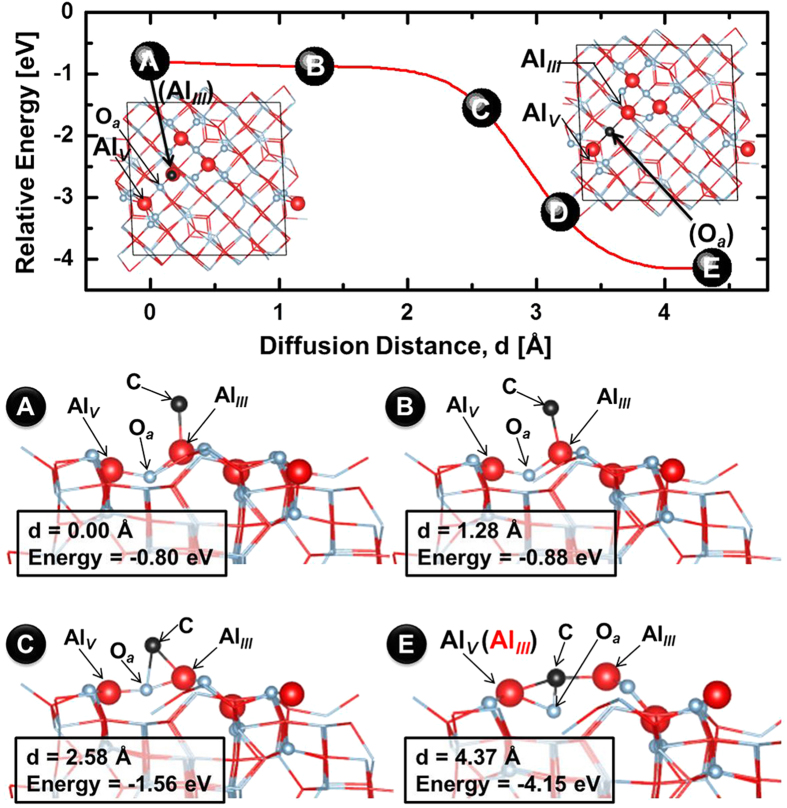
Minimum energy path for a generated C adatom from the initial Al_*III*_ site (A) to the final O_*a*_ site (E). The red curve guides the eye and was fit through the eight points spaced by 0.63 Å that were calculated using a climbing image nudged elastic band model. Among these, five are marked with filled circles, while side-view snapshots along the minimum energy path are shown for four. The inset images are top-views from the initial to the final site of the C adatom. The diffusion distance, d, and the relative energy of the C adatom are shown in each snapshot.

To obtain E_d_, we calculated the energy path from the Al_*III*_ site to its adjacent O_*a*_ site using the climbing image nudged elastic band (CI-NEB) model^37^,^38^. As shown in Fig. 3, the minimum energy path predicted in this way indicates that the associated barrier is negligible (E_d_ ≈ 0 eV). This contrasts with the values obtained for Ag and Ni, (viz. 0.20 eV and 0.39 eV, respectively) but is comparable to the barrier on Cu (0.07 eV)^39^. To confirm this result, we considered the energies associated with the formation of C dimers involving an adatom. The results calculated in atom-by-atom mode, presented in Figure S7, show that the formation of a C dimer on the O_*a*_ site (C-C-O_*a*_) is the most stable reaction, with a relative energy of 6.06 eV per C atom. No surface diffusion barriers were evidenced in this process, and since the formation of a bridging-metal linear C_n_-O_*a*_ structure (n is the number of C atoms) is similar to the formation of graphene nuclei on α-Al_2_O_3_^10^, these results also show that E_d_ for the latter is negligible.

The enlargement of graphene nuclei proceeds according to E_att_. A value of 0.21 eV was obtained using a simple model in which the growth of graphene islands is governed by the edge controlled kinetics of the attachment and detachment of C adatoms, as described on page S26 in the SI.

### Interpretations of growth phenomena related with material characteristics

Table 1 lists the various surface activation barriers, C solubility, activation barrier for nucleation, and growth mechanisms associated with different growth materials. Ignoring E_d_ and C solubility, Ni appears at first glance to be the most favorable substrate. However, these two terms can lead to the growth of graphene on Ni substrates both by segregation and precipitation.

The supply of C adatoms is governed by E_ad_, while as mentioned above, E_att_ governs the enlargement of the graphene nuclei. Therefore, these two activation barriers determine the lowest possible growth temperature. For the theoretical calculation of E_c_, Kim *et al*.^35^ used an E_att_ of 2 eV for Cu, based on growth experiments performed on Ru^36^. This approximation notwithstanding, regardless of the value used for E_att_ (either 2 eV or 0.2 eV from DFT calculation for other transition metals^39^), the value for E_c_ they calculate does not match their own experimental measurement (E_c_ = 1 eV)^35^. Using the equation derived here however, as shown at Table 1, an E_att_ of 0.2 eV yields E_c_ = 1.1 eV, demonstrating that this equation, originally derived for metal oxides, is also valid for metal catalysts. One also notes that the DFT-calculated value for E_att_ (0.2 eV for transition metals) is similar to the one measured here (0.21 eV). In this context, E_ad_ has a significant impact on the catalytic performance of the substrates. Since E_ad_s is similar for γ-Al_2_O_3_ and Ni, the growth temperature of graphene on γ-Al_2_O_3_ should be comparable to that on Ni^16^. Finally, since E_d_ determines the homogeneity of the graphene layer grown on the substrate, and since E_d_ is the lowest in γ-Al_2_O_3_, this should be the most favorable of these three substrates for the formation of homogeneous graphene. A further discussion of C solubilities and a quantitative comparison of the different substrates may be found on page S28 of the SI.

### Characterization of graphene grown on γ-Al_2_O_3_

**Figure 4:**
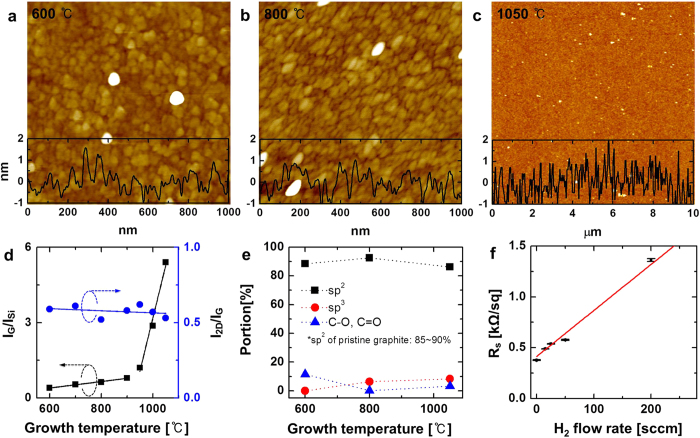
Characteristics of the grown graphene. Atomic force micrographs obtained from graphene samples grown on γ-Al_2_O_3_ substrates at **a**, 600 °C, **b**, 800 °C, and **c**, 1050 °C. **d**, shows the variation with the growth temperature of the I_2D_/I_G_, I_G_/I_Si_ Raman peak intensity ratios. **e**, Variation as a function of the growth temperature of the proportion of the total integrated intensity accounted for by sp^2^, sp^3^, C-O, and C=O peaks in the C 1s region of X-ray photoelectron spectra. **f**, Sheet resistance (R_s_) plotted as a function of the H_2_ flow rate. The red dotted line is provided to guide the eye.

Atomic force microscopy (AFM) images of graphene synthesized for 1 h at 600 °C, 800 °C, and 1050 °C, are respectively shown in Figs 4a–c. Nanometer-scale grains are observed in the samples grown at 600 °C and 800 °C, but no grain boundaries are visible in the sample grown at 1050 °C. The analysis of the AFM images is summarized in Table S1. Raman spectra obtained from the same samples are shown in Figure S10 and the parameters resulting from this analysis are listed in Table S2. Figure 4d plots the intensity ratio of the G peak to the Si peak (*I*_*G*_/*I*_*Si*_), and of the 2D peak to the G peak (*I*_*2D*_/*I*_*G*_), as a function of the growth temperature. The latter ratio reports on the number of graphene layers formed, with a values of ~0.5 at an excitation wavelength of 532 nm indicating the presence of bilayers. Figure 4d shows thereby that the graphene obtained here is bilayered, regardless of the growth temperature. On the other hand, *I*_*G*_/*I*_*Si*_ reports on the average area of the graphene grains. The variation of this intensity ratio with the growth temperature follows two distinct slopes in Fig. 4d, revealing two different mechanisms for grain growth, as also shown in Fig. 1f and Figure S4.

We also analyzed our graphene samples by using X-ray photoelectron spectroscopy (XPS) and X-ray diffraction (XRD) to identify the crystalline phase of the Al_2_O_3_ substrate and evaluate the quality of the graphene. High-resolution XPS profiles were acquired in the C 1s (see Figure S12 in the SI), Al 2p, O 1s, and N 1s (Figure S13) regions to characterize the graphene, the γ-Al_2_O_3_ substrate, and the N-doping in the graphene sheets (see page S33 of the SI). Figure 4e shows the evolution of the relative contents of different C components in the graphene as a function of the growth temperature. The proportion of sp^3^ carbon increases at higher temperatures while that of oxygen-bonded carbon moieties decreases. The sp^2^ content remains constant at ~90%. The total carbon content in the graphene sheets is greater than 97%, which is slightly superior even to that of pristine graphite (~96%)^40^. The reader is referred to page S36 and Figure S14 in the SI for the corresponding XRD analysis.

Figure 4f shows the sheet resistance (R_s_) of the graphene samples as a function of the H_2_ flow rate used during CVD (under a constant 850 sccm flow of CH_4_). The linear increase of R_s_ with the H_2_ flow observed here can be expressed by the following equation.


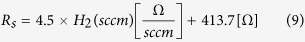


H_2_ is the flow from the mass flow controller (sccm).

The optimal H_2_ flow rate was also investigated by Raman spectroscopy, by looking at its influence on the *I*_*D*_/*I*_*G*_ ratio (see Figure S11). This analysis also shows that graphene synthesis is best conducted under H_2_-free conditions, or with minimal H_2_ flow (~25–50 sccm), a strong argument in favor of the use of γ-Al_2_O_3_ as a growth substrate. It is noteworthy moreover that the R_s_ standard deviation is less than 1% in all the samples prepared with H_2_ flows up to 200 sccm (see Figure S11c). In other words, the optimal samples prepared here have a R_s_ of ~0.4 kΩ/sq with less than 1% standard deviation, a performance comparable or superior to that of graphene grown on Cu^22^.

**Figure 5:**
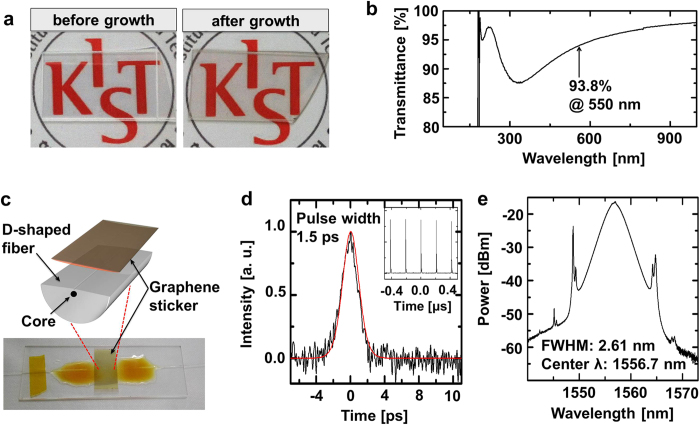
Linear and nonlinear optical characteristics of graphene grown on γ-Al_2_O_3_. **a**, Photographs of the transparent substrates (Al_2_O_3_ 50 nm/quartz wafer/Al_2_O_3_ 50 nm) before and after graphene growth. **b**, The optical transmittance of the graphene sample as a function of wavelength. The transmittance is 93.8% at 550 nm. **c**, A schematic diagram and a photograph showing the attachment of a graphene sticker to a D-shaped fiber, the main component of ultrafast mode-locked lasers. **d** and **e** are the measured output pulses of the autocorrelation trace (Inset: the pulse waveforms) and the optical spectrum, respectively. The autocorrelation was analysed by assuming a transform-limited sech^2^ pulse (red line in **d**).

Optical transmittance measurements are a reliable means to determine the number of graphene layers synthesized. For these experiments, we synthesized graphene onto Al_2_O_3_/Quartz wafer/Al_2_O_3_ substrates as shown in Fig. 5a, with the resulting optical transmittance spectrum shown in Fig. 5b.

The optical transmittance of graphene, T_opt_, can also be obtained from Lee’s equation^41^,


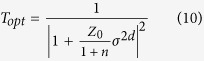


in which Z_0_ is the free-space impedance (377 Ω) and n is the refractive index of the Al_2_O_3_ substrate (1.77 at 550 nm). According to this equation, a single layer of graphene grown on Al_2_O_3_ absorbs ~1.66% of the incident light. As shown in Fig. 5b, the value measured here for T_opt_ at 550 nm is 93.8%, which matches the value (93.76%) predicted using Equation (10) for graphene bilayers grown on the top and bottom face of an Al_2_O_3_/Quartz wafer/Al_2_O_3_ substrate^22^. The performance of these devices and the bending tests and Hall measurements performed thereon have been reported elsewhere^22^.

As a practical application of our readily detachable graphene layers, we incorporated one of these into an ultrafast mode-locked laser^2^,^3^, as shown in Fig. 1a,c. The graphene layer was manually transferred to the nonlinear photonic device as a graphene sticker, as shown in Fig. 5c. The graphene acted as a nonlinear saturable absorber to produce ultrafast laser pulses in a fiber ring cavity (see Figure S15)^3^ by inducing a passive mode-locking of the co-propagating multiple sub-modes. Fig. 5d shows the autocorrelation trace of the successfully pulsed laser output with a pulse duration of 1.5 ps. The inset shows the pulse train obtained with a repetition frequency of 4.8 MHz. Figure 5e shows the corresponding optical spectrum, with a central wavelength of 1556.7 nm and a full width at half maximum of 2.61 nm.

In summary, the use of catalytic γ-Al_2_O_3_ as a substrate for the highly efficient formation of graphene has been explored and the entire formation mechanism has been elucidated using both numerical simulations and experiments. This synthesis route is effective at low temperatures and without metal, and allows grain size customization and the preparation in a single step of graphene/dielectric layer structures. Moreover, the graphene produced in this way is uniform over large areas in terms of its electrical properties and is readily detachable.

We evaluated theoretically the activation energies associated with all the stages of graphene nucleation and growth, confirming that the low energy barriers for the surface diffusion of C adatoms and for graphene nucleation/growth on the γ-Al_2_O_3_ substrate ensure the synthesis of high quality graphene. Finally, we derived a universal equation, which describes the adsorption-based synthesis of graphene over a wide temperature range (with catalytic and spontaneous growth at low and high temperatures, respectively) and on a number of different substrates, including metal catalysts. The experimental results obtained in this study support the proposed equation.

To demonstrate the potential practical applications of this synthesis route, we manually transferred one of the ‘graphene stickers’ prepared in this way to fabricate an ultrafast mode-locked laser.

## Methods

### Formation of the γ-Al_2_O_3_ substrates

The γ-Al_2_O_3_ substrates (50 nm thick) were synthesized via CVD for the growth of graphene on amorphous ALD Al_2_O_3_ substrate with 850 sccm of CH_4_ and 50 sccm of H_2_ at 1050 °C. In order to get the γ-Al_2_O_3_ substrate, the graphene grown was detached by using a polyimide film^22^.

### Graphene synthesis

Except for the optimization of the gas flow rates, all the graphene samples were synthesized using 850 sccm of CH_4_ and 50 sccm of H_2_, with different growth temperatures and durations.

### Graphene synthesis on transparent substrates for transmittance measurements

Al_2_O_3_ (50 nm thick) was synthesized via ALD onto a quartz wafer coated on its backside with a polymer film. The backside was also coated with Al_2_O_3_ using the same method. Graphene was then synthesized on both sides of the Al_2_O_3_/wafer quartz/Al_2_O_3_ substrate for 15 min at 1050 °C.

### Graphene stickers and graphene mode-lockers

Graphene stickers were prepared by detaching spin-coated polyimide together with the graphene from γ-Al_2_O_3_ substrates. A D-shaped optical fiber was fabricated by polishing one side of a conventional single-mode fiber (SMF) fixed with epoxy on a slide glass^3^. The graphene sticker was simply tailored with scissors and attached onto the D-shaped fiber as shown at Figs 1c and 5c. A graphene mode-locked laser with a fiber ring cavity was prepared as described previously^3^. A home-made erbium-doped fiber amplifier was used as a gain medium to generate an average output power of 5.1 dBm. An additional 20-m long SMF was used to optimize the intracavity chromatic dispersion. A polarization controller was added to match the roundtrip polarization state in the cavity, into which isolators were inserted to guarantee unidirectional light propagation. A 90/10 coupler was employed for the output of the pulsed laser.

### Characterization

Atomic force micrographs were acquired in non-contact mode with a ppp-NCHR 5M probe (Nanosensors) attached to a XE-100 microscope (Park Systems). The Raman spectra were acquired using a Renishaw In-Via system with a 532 nm excitation laser. The XRD profiles were obtained using a Dmax2500/PC (Rigaku) spectrometer operated at 40 kV, 200 mA, and 8 kW using a Cu target (1.5406 Å) at a scan rate of 2° min^−1^. The XPS profiles were acquired using a PHI 5000 VersaProbe (Ulvac-PHI) system at a base pressure of 6.7 × 10^−8^ Pa using a monochromated Al Ka (1486.6 eV) anode (25 W, 15 kV) with a spot size of 100 μm × 100 μm. The transmittance data were recorded on a Cary 5000 (Varian) spectrometer from 175 nm to 3300 nm at a scan rate of 600 nm·min^−1^ at a resolution of 1.0 nm. The optical spectrum, its characteristics, and those of the pulse waveform, were respectively measured using an autocorrelator (25 fs resolution, HAC-200, Alnair Labs), an optical spectrum analyzer (0.02 nm resolution, C-band scan range, SW7370C, Yokogawa), and an oscilloscope (DSO 5054A, Agilent Technology).

### Computational methods

Calculations were performed using the Vienna ab initio Simulation Package^42^. The projector augmented wave method^43^ was used with a cut-off energy of 500 eV, within the generalized gradient approximation parameterized by Perdew and Wang (PW91)^44^. The 3s and 3p orbitals of Al and the 2s and 2p orbitals of C and O were treated as the valence electrons in the spin-polarized calculations. The optimization of bulk γ-Al_2_O_3_ was carried out following the method proposed by Digne, *et al*.^30^. The (110) surface of the conventional cubic spinel structure corresponds to the (001) surface optimized by Digne, *et al*.^30^. The Al_2_O_3_ slab with eight layers was reconstructed after having been cut to a size of 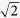 ×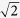, and rotated by an angle of 45° with 15 Å vacuum. The number of Al and O atoms was 64 and 96, respectively. The surface was a 11.65 Å × 11.65 Å square slightly tilted at an angle of 92.30°. The 3 × 3 × 1 Γ-centred k-grid was used. Dipole corrections were applied according to the c-axis direction.

The adsorption energy of a C atom on the Al_2_O_3_ surface was defined as follows.


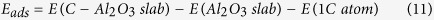


The energy of one C atom was calculated in a 20 Å × 20 Å × 20 Å vacuum space. A CI-NEB calculation was performed to calculate the migration barrier energy of the attached carbon^37^,^38^.

C atom adsorption was tested on top of the Al_*III*_, O_*a*_, O_*b*_, and O_*c*_ sites. The Al_*III*_ site, which has three O neighbors, is reported to act as a catalytic site for the dissociation of CH_4_^28^,^29^. The O_*a*_, O_*b*_, and O_*c*_ sites are defined as O atoms having bonds with Al_*III*_ and Al_*V*_, with Al_*III*_ and left-Al_*IV*_ (Al_*IV*_ (l)), and with Al_*III*_ and right-Al_*IV*_ (Al_*IV*_ (r)), respectively.

## Additional Information

**How to cite this article**: Park, J. *et al*. Growth, Quantitative Growth Analysis, and Applications of Graphene on γ-Al_2_O_3_ catalyst. *Sci. Rep*. **5**, 11839; doi: 10.1038/srep11839 (2015).

## Supplementary Material

Supplementary Information

## Figures and Tables

**Table 1:** Comparison of the activation barriers governing graphene growth on different substrates.

	E_ad_ [eV]	E_d_ [eV]	E_des_ [eV]	E_att_[eV]	C solubility [at%]	E_c_ of N_s_[eV]	E_a_ of J [eV]	Growth mechanism
γ-Al_2_O_3_	0.88	0	4.15	0.21	NONE^a^)	0.21^f^)	6.96^g^)	Surface
						0.22^g)^		adsorption
								
Cu	3.4~4.1^45^	0.06~0.7^46^	6^45^	0.2^d^),^39^	0.001^a^^),32^	1^f^)^39^	19.2^g^)	Surface
				2^e^),^35^,^36^		1.1^d^),^g^), 0.46^e^),^g^)		adsorption
Ni	0.74^b^,^47^	0.37^b^),^48^,^49^	6.28^b^),^49^	0.2^39^	1.3^a^),^32^	0.05^g)^	11.08^g^)	Segregation or precipitation
			6.76^c^),^49^					

^a^at 1000 °C.

^b^(111) surface.

^c^(110) surface.

^d^estimated from results for other transition metals.

^e^estimated from results for Ru.

^f^estimated by experiment.

^g^estimated using the proposed growth model.
