# Supplementary material for: Growth, Quantitative Growth Analysis, and Applications of Graphene on γ-Al2O3 catalysts
Source: Sci Rep. 2015 Jul 3;5:11839. doi: 10.1038/srep11839 (PMC4490347; doi:10.1038/srep11839)
Supplement: Supplementary Information [file srep11839-s1.doc]

**Supplementary Information**

**Growth, Quantitative Growth Analysis, and Applications of**

**Graphene on γ-Al_2_O_3_ catalyst**

*Jaehyun Park^1^, Joohwi Lee^2^, Jung-Hae Choi^2^, Do Kyung Hwang^1^, and Yong-Won Song^1^,**

1. Center for Opto-Electronic Materials and Devices, Korea Institute of Science and Technology, Hwarangno 14-gil 5, Seongbuk-gu, Seoul 136-791, Republic of Korea
2. Center for Electronics Materials, Korea Institute of Science and Technology

Hwarangno 14-gil 5, Seongbuk-gu, Seoul 136-791, Republic of Korea

*E-mail: [ysong@kist.re.kr](mailto:ysong@kist.re.kr)

**Analysis of the growth behaviours of graphene nuclei**

We have taken the relative nuclei areas dependent on the growth temperatures in the form of the *I_G_*/*I_Si_* ratios of Raman spectra, as depicted in Figure 4d (Figure S1a). Therefore, the relative densities of the nuclei as a function of the growth temperatures are proportional to 1/(*I_G_*/*I_Si_*). To analyse the growth behaviours of the graphene nuclei, we used the Arrhenius equation for evaluating the growth activation barriers by using the natural logarithm of the density of the nuclei vs. 1/T, and the results are shown in Figure S1b. There were two distinct growth regimes, the one is from 600 °C to 900 °C and the other is from 950 °C to 1050 °C. Each growth activation barrier was evaluated by using the linear slope, which was divided by the Boltzmann constant, k = 8.62 × 10^-5^ eV k^-1^. The steep slope is the activation barrier of the spontaneous dehydrogenation of CH_4_, which is denoted as E_s_ = 2.10 eV, and the less steep slope is the activation barrier for the catalytic dehydrogenation, denoted as E_c_ = 0.21 eV.


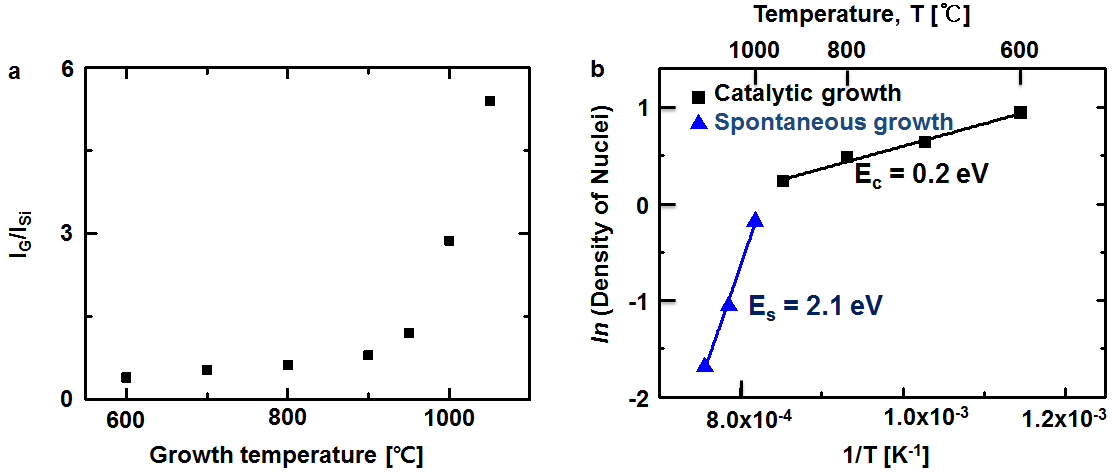


**Figure S1.** To evaluate the activation barriers of the graphene nuclei, **a**, the relative areas as a function on the growth temperatures were plotted in the form of the *I_G_*/*I_Si_* of the Raman spectra. **b**, The Arrhenius plot of the natural logarithm of the density of the graphene nuclei, *ln*(Density of Nuclei) vs. 1/T.

**Numerical derivation of graphene formation and growth**

To evaluate the theoretical growth mechanism of the two distinct growth regimes shown in Figure 1g and Figure S1, we used the Robinson and Robins model^1^. This model describes the formation and enlargement of nuclei from physically evaporated atoms by considering the capture, surface diffusion, and re-evaporation of the adatoms. Since these factors are insufficient for describing graphene nucleation behaviours, we created a scheme of energy landscape, consisting of various activation barriers related with the dissociative adsorption of CH_4_, surface diffusion of C adatoms, desorption of C adatoms, and the detachment and attachment of C adatoms from/to the graphene nuclei, as shown in Figure 1f.

**Kinetic theory of gases**

As the pressure of our thermal chemical vapour deposition (CVD) is ~4 torr, we assumed CH_4_ and H_2_ as ideal gases.

The number density of gases, ρ, is given by the following equation.

$\rho=\frac{p}{kT}$ (S1)

p is the partial pressure and k is the Boltzmann constant.


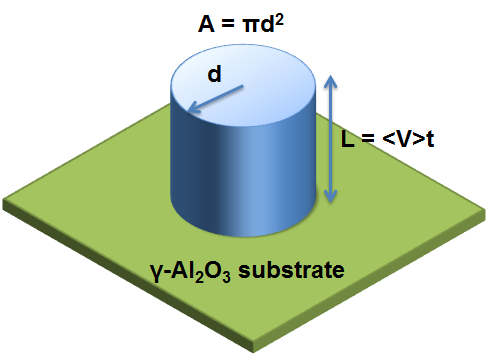


**Figure S2.** An imaginary cylinder consisting of CH_4_ on the γ-Al_2_O_3_ substrate. Its cross sectional area is A and height is L.

Let us assume an imaginary cylinder of cross sectional area A and height L. A as shown at Figure S2 is related to the diameter d by the following equation.

$A=\pi d^{2}$ (S2)

$L=\langle V\rangle t$ (S3)

In the above equation, <V> is the average velocity of the gas and t is the time.

$\langle V\rangle=\sqrt{\frac{8kT}{\pi m}}$ (S4)

In Equation (S4), m is the mass of the molecule.

Then, the collision frequency, Z, is related to the < V > by the following equation.

$Z=\sqrt{2}\rho A\langle V\rangle$ (S5)

Consequently, the molecular flux, i.e. the number of molecules striking the γ-Al_2_O_3_ substrate per unit area per unit time, F, is given by Equation (S6).

$F=(\frac{1}{4})\rho\langle V\rangle$ (S6)

$F=\frac{p}{\sqrt{2\pi mkT}}$ (S7)

Therefore, the impinging rate of the molecule on the γ-Al_2_O_3_ substrate, r, can be given by the following equation.

$r=Fs_{0}$ (S8)

In the above equation, s_0_ is the initial sticking coefficient on the substrate.

In our CVD system, the C precursor is CH_4_, which should pass the dissociative adsorption barrier, E_ad_, to be a C adatom.

Therefore, the dissociative adsorption probability is .

Consequently, the impinging rate of the C adatoms from CH_4_ molecules, R, can be expressed as follows.

$R=\frac{p_{CH_{4}}}{\sqrt{2m_{CH_{4}}kT}}s_{0}\exp^{(-\frac{E_{ad}}{kT})}$ (S9)

**Probability equations**

The surface diffusion paths of the generated C adatoms from the Al*_III_* site to the pre-designated sites may probably depend on the activation barriers of surface diffusion, attachment, and re-evaporation. The probabilities are related to the activation barriers. Taking E_d_ as the diffusion barrier of the C adatom to form and to grow the graphene nuclei and E_des_ as the desorption barrier of the C adatom during the surface diffusion, the probabilities of diffusion, p_d_, and desorption, p_des_, of the C adatom are expressed as follows.

$p_{d}=\upsilon_{0}\exp^{(-\frac{E_{d}}{kT})}$ (S10)

$p_{des}=\upsilon_{0}\exp^{(-\frac{E_{des}}{kT})}$ (S11)

In the above equations, ν_0_ is the nth order vibrational frequency (= 10^13^ s^-1^ in most cases).

The reciprocal of E_des_ is the lifetime (τ) of the adsorbed adatom on the surface expressed as follows.

(S12)

E_att_ is the attachment barrier of the C adatom at the edge of the graphene nuclei and E_m_ is the barrier to the coalescence of the nuclei. The probability of attachment, p_att_, and the probability of coalescence, p_m_, can be expressed as follows.

$p_{att}=\upsilon_{0}\exp^{(-\frac{E_{att}}{kT})}$ (S13)

and

$p_{m}=\upsilon_{0}\exp^{(-\frac{E_{m}}{kT})}$ (S14)

**Rate equations**

The nucleation density can be derived from the rate equations of the population densities. The population densities of single C adatoms, a pair C adatoms, and that of graphene nuclei consisting of x adatoms (N_1_, N_2_, and N_x_, respectively) were used to obtain the rates R, R_2_, and R_x_, respectively. For diffusion, the activation barrier of desorption should be larger than that of diffusion. Occasionally, the magnitude of E_des_ was assumed to identical to that of E_ad_ in the simulations. In such cases, the adsorption site should be equal to the desorption site. However, catalysts involved in the formation of graphene, such as Cu and Ni, have different magnitudes of E_ad_ and E_des_, as shown in Table 1. The reason is that the magnitude of E_des_ is significantly larger than E_d_. This feature causes the surface diffusion before the desorption of C adatoms occurs. This kind of surface diffusion can be happen more easily on the γ-Al_2_O_3_ substrate because of the negligible value of E_d_, low anchoring or attachment barrier, and large stabilization energies of the minimum energy path for destination sites shown in Figure 3, Figure S5, Figure S6 and Figure S7. Therefore, the E_des_ should be defined on an O*_a_* site, which is the most prominent site for the anchoring of the C adatom, as shown in Figure 3 and Figure S5. Subsequently, the E_des_(C-O*_a_*) is 4.15 eV (assuming that the desorbed C adatom is a part of R_2_ and R_c_).

$C(ad)/O_{a}(s)\to C(g)+O_{a}(s)$ (S15)

The attachment of additional C adatoms to form N_2_ is also expected such that it does not have any attachment and diffusion barriers, as shown in Figure 2b. Consequently, we assumed that the attachment barrier of small nuclei that consist of small numbers of C adatoms is E_att,s_ and those of large nuclei is E_att_. The detachment energy barriers of N_2_ and N_x_ are 6.06 eV and 9.24 eV (graphene) from our DFT calculations. We neglected the detachment of C adatoms from the nuclei because of the large activation barrier in view of the CVD conditions used.

We also assume that the desorption of C adatoms is a part of the rate of pair formation, R_2_ and the rate of N_x_ formation, R_c_. Therefore, the balance rate equation from N_1_ can be expressed as follows.

$dN_{1}/dt=R-2R_{2}-R_{c}$ (S16)

R_2_, is expressed as follows

$R_{2}=Cp_{d}p_{att,s}N_{1}^{2}/N_{0}$ (S17)

where, C is based on the number of effective pair-formation sites neighbouring any single adatom and p_d_p_att_(or p_att,s_)N_1_ is the frequency at which sites are visited by the diffusion of adatom.

Similarly, R_c_ for the rate of the nuclei growth is expressed in the following equation.

$R_{c}=q_{0}p_{d}p_{att}N_{1}N_{x}/N_{0}$ (S18)

The coalescence rate, R_m_, for the rate of the coalescence of the C adatoms by the nuclei is given by the following equation.

$R_{m}=\beta p_{d}p_{m}N_{1}N_{x}^{2}/N_{0}^{2}$ (S19)

Both R_c_ and R_m_ receive contributions from the migration of the C adatoms. However, the attachment barrier of the migrated C adatoms changes in magnitude during the coalescence because the adatoms interact with two or more nuclei boundaries. Therefore, p_att_ is re-defined as p_m_ to reflect the changes in the attachment barriers.

From these equations, we obtained the following highly non-linear differential equations similar to the Robinson and Robins model^1^.

$\frac{dN_{x}}{dt}=\frac{Cp_{d}p_{att,s}N_{1}^{2}}{(1-p_{m})N_{0}}-\frac{\beta p_{d}p_{m}N_{1}N_{x}^{2}}{N_{0}^{2}}=\frac{Cp_{d}p_{att,s}N_{1}^{2}}{N_{0}}-\frac{\beta p_{d}p_{m}N_{1}N_{x}^{2}}{N_{0}^{2}}$ (S20)

In the above equation, β is an undetermined dimensionless coefficient.

(1-p_m_) indicates the additional rate of R_2_ due to the coalescence of the nuclei. However, as p_m_ is neglected with respect to 1, 1+p_m_ can be easily reduced to 1.

To obtain the solution, the ratio, η, was used as a determinant for the growth regime.

$\eta=\frac{N_{0}p_{des}}{q_{0}p_{d}}$ (S21)

In the above equation, N_0_ is the density of adsorption sites in the surface and q_0_ is a dimensionless coefficient representing an appropriate average over the population. This determinant is highly dependent on the validity of the magnitude of the two activation barriers E_des_ and E_d_ under the growth temperatures used.

Robinson and Robins^1^ set two kinds of growth regimes, one is the high temperature limit (N_x_ « η) and the other is the low temperature limit (N_x_ » η). Kim et al^2^. refer the former as the desorption controlled regime and the latter as the capture controlled regime. We will refer the former and latter as the high temperature and catalytic growth regimes, respectively. The transition temperature between the spontaneous growth and the catalytic growth of graphene nuclei is related to the dehydrogenation temperature of CH_4_ on γ-Al_2_O_3_. We believe that the growth temperature of ~950 °C is the universal growth temperature for forming graphene on any substrate, which should be able to withstand this growth temperature. For forming graphene below 900 °C, the growth substrates should possess catalytic activities to decompose the CH_4_ molecules to C atoms. Moreover, the growth tendency fits well with the results predicted by Robinson and Robins^1^.

From Equation (S18), the following equation can be derived.

$N_{1}=\frac{R_{c}N_{0}}{q_{0}p_{d}p_{att,s}N_{x}}$ (S22)

During the initial growth or flash growth, N_1_ will increase abruptly. As the growth proceeds, R_c_, which is used for the areal expansion of nuclei becomes R. Therefore, the following equation can be constructed.

$N_{1}=\frac{RN_{0}}{q_{0}p_{d}p_{att}(\eta+N_{x})}$ (S23)

In Equation (S20), N_1_ is substituted by Equation (S23) to obtain the following equation.

$(\eta+N_{x})^{2}\frac{dN_{x}}{dt}=\frac{CR^{2}p_{att,s}N_{0}}{q_{0}^{2}p_{d}p_{att}^{2}}-\frac{\beta p_{m}R}{q_{0}p_{att}N_{0}}(\eta+N_{x})N_{x}^{2}$ (S24)

At the high temperature growth regime, η + N_x_ ~ η and at this regime, Equation (S24) can be reduced to the following equation.

$\frac{dN_{x}}{dt}=\frac{N_{s}^{2}-N_{x}^{2}}{\tau N_{s}}$ (S25)

In the above equation, $N_{s}^{2}=\frac{CRP_{att,s}N_{0}}{\beta p_{des}p_{att}p_{m}}$ and the solution is N_x_ = N_s_ tanh(t/τ).

Therefore,

$N_{s}=(\frac{CN_{0}}{\beta\upsilon})^{\frac{1}{2}}R^{\frac{1}{2}}\exp(\frac{E_{des}+E_{attt}+E_{m}-E_{att,s}}{2kT})$ (S26)

$N_{s}=(\frac{CN_{0}}{\beta\upsilon})^{\frac{1}{2}}(\frac{p_{CH_{4}}}{\sqrt{2m_{CH_{4}}kT}}s_{0})^{\frac{1}{2}}\exp(\frac{E_{des}+E_{attt}+E_{m}-E_{att,s}-E_{ad}}{2kT})$ (S27)

As the solution is a function of tanh(t/τ), the activation barrier of the high temperature growth regime, E_h_ can be expressed as follows.

$E_{h}=\frac{E_{des}+E_{att}+E_{m}-E_{att,s}-E_{ad}}{2}$ (S28)

At the catalytic growth regime, η + N_x_ ~ N_x_ and at this regime, Equation (S24) can be reduced to Equation (S29).

$\frac{dN_{x}}{dt}=\frac{N_{s}^{3}-N_{x}^{3}}{\tau}$ (S29)

In the above equation, $N_{s}^{3}=\frac{CRP_{att,s}N_{0}^{2}}{\beta q_{0}p_{d}p_{att}p_{m}}$ and the solution is$N_{x}=N_{s}\{1-\exp(-t/\tau)\}^{1/3}$.

Therefore,

$N_{s}=(\frac{CN_{0}^{2}}{\beta q_{0}\upsilon})^{\frac{1}{3}}R^{\frac{1}{3}}\exp(\frac{E_{d}+E_{attt}+E_{m}-E_{att,s}}{3kT})$ (S30)

$N_{s}=(\frac{CN_{0}^{2}}{\beta q_{0}\upsilon})^{\frac{1}{3}}(\frac{p_{CH_{4}}}{\sqrt{2m_{CH_{4}}kT}}s_{0})^{\frac{1}{3}}\exp(\frac{E_{d}+E_{attt}+E_{m}-E_{att,s}-E_{ad}}{3kT})$ (S31)

As the solution is a function of exp(-t/τ)^1/3^, the activation barrier of catalytic growth regime, E_c_ can be expressed as follows.

$E_{c}=\frac{E_{ad}+E_{att,s}-E_{att}-E_{d}-E_{m}}{3}$ (S32)

**Transition temperature (T_c_) between the catalytic growth regime and high temperature growth regime**

It is very challenging to define the transition range between the catalytic and spontaneous growth temperature. However, T_c_ in the middle of transition range can be found by the N_s_ of the catalytic growth, which is equal to the that of the high temperature growth as expressed below.

$(\frac{CN_{0}}{\beta\upsilon})^{\frac{1}{2}}(r)^{\frac{1}{2}}\exp(\frac{E_{des}+E_{attt}+E_{m}-E_{att,s}-E_{ad}}{2kT_{c}})=(\frac{CN_{0}^{2}}{\beta q_{0}\upsilon})^{\frac{1}{3}}(r)^{\frac{1}{3}}\exp(\frac{E_{d}+E_{attt}+E_{m}-E_{att,s}-E_{ad}}{3kT})$ (S33)

$T_{c}=\frac{3E_{des}+E_{att}+E_{m}-E_{att,s}-E_{ad}-E_{d}}{k\ln(\frac{\beta\nu N_{0}}{Cq_{0}^{2}r})}$ (S34)

At this temperature, *N_0_ = q_0_* , $\frac{\beta\nu N_{0}}{Cq_{0}^{2}r}=\upsilon\frac{R_{m}N_{1}^{2}p_{d}^{2}p_{att,s}^{2}}{R_{2}R_{c}r}$.

Let R_m_ = R_c_^2^. Hence,

$\upsilon\frac{R_{m}N_{1}^{2}p_{d}^{2}p_{att,s}^{2}}{R_{2}R_{c}r}=\upsilon\frac{R_{c}(N_{1}^{2}p_{d}^{2}p_{att,s}=R_{2})}{R_{2}r}=\upsilon\exp(Ead).$ (S35)

Therefore,

$T_{c}=\frac{3E_{des}+E_{att}+E_{m}-E_{att,s}-E_{ad}-E_{d}}{k\ln(\upsilon\exp(E_{ad}))}\approx4800K.$ (S36)


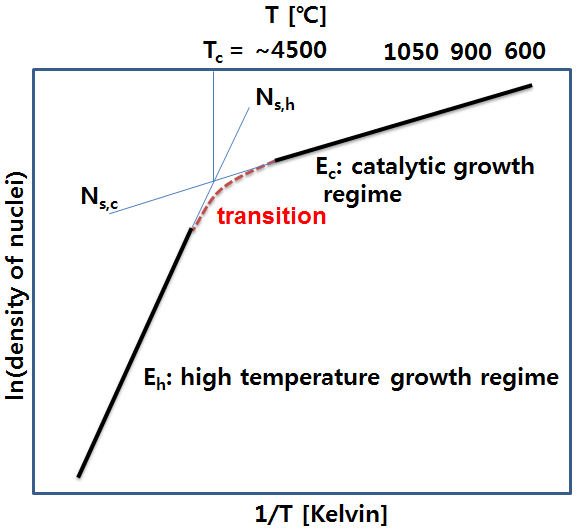


**Figure S3.** Schematic Arrhenius plot of the natural logarithm of the saturation density of nuclei N_s_, ln(density of nuclei), vs. 1/T. N_s, c_ and N_s, h_ indicate the N_s_ at the catalytic growth regime and that at high temperature growth regime, respectively. Their intersection point T_c_ indicates the transition temperature calculated from Equation (S36).

**The spontaneous dehydrogenation regime based on the catalytic growth regime**

From the above result, T_c_ as shown at Figure S3 is significantly above the growth temperatures ranging from 950 °C to 1050 °C. Therefore, it is challenging to apply the result from the high temperature growth regime to the growth behaviours under this range of temperatures. Therefore, we considered the overall growth behaviour based on the catalytic growth regime and the difference is the number of adsorption sites related with the growth temperature, which would affect the dissociative adsorption rate of CH_4_ (see Figure S4).

The number of initial adsorption sites is q_0_ and the final number of adsorption sites is N_0_. This change can be defined with the Boltzmann distribution for the catalytic activation, according to the growth temperature.

During the growth of graphene by using CH_4_ above 900 °C, the saturation density, N_s_′, can be defined by considering the number of adsorption sites as follows.

$R'=(\frac{q_{0}}{N_{0}})\exp(\frac{-1}{kT})R=(\frac{p_{CH_{4}}}{\sqrt{2m_{CH_{4}}kT}}s_{0})^{\frac{1}{2}}\exp(\frac{-E_{ad}-3\ln(\frac{q_{0}}{N_{0}})}{3kT})$ (S37)

$N_{s}'=(\frac{CN_{0}^{2}}{\beta q_{0}\upsilon})^{\frac{1}{3}}(\frac{p_{CH_{4}}}{\sqrt{2m_{CH_{4}}kT}}s_{0})^{\frac{1}{2}}\exp[\{(3\ln(\frac{q_{0}}{N_{0}})+(E_{ad}+E_{att,s}-E_{att}-E_{d}-E_{m})\}/3kT]$ (S38)

Therefore, the E_a_s of each growth regimes are given by equations (S39) and (S40), respectively.

$E_{c}=\frac{E_{ad}+E_{att,s}-E_{att}-E_{d}-E_{m}}{3}$ (S39)

$E_{s}=\frac{E_{ad}+E_{att,s}-E_{att}-E_{d}-E_{m}}{3}+\ln(\frac{q_{0}}{N_{0}})$ (S40)

For evaluating E_att_, we measured the areal distributions of the graphene nuclei by AFM. It was difficult to expect the existence of E_att,s_ because of the large difference (of ~1.91 eV) between the stabilization energies of the C monomer and the C dimer. Therefore, we concluded that E_att_ – E_att,s_ is E_att_. Moreover, we could neglect E_m_ (See page S19, SI). Finally, Equations (S39) and (S40) can be simplified in to equations (1) and (2), respectively.

Therefore, E_c_ and E_s_′are 0.22 eV and 2.09 eV, respectively.


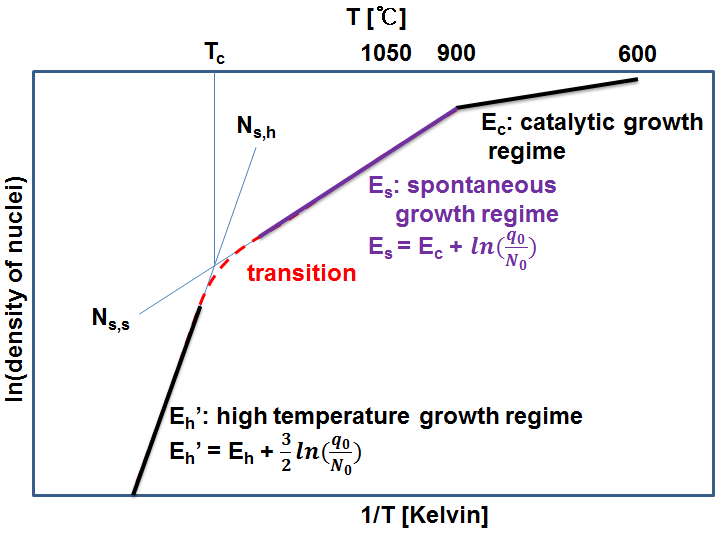


**Figure S4.** The Arrhenius plot of ln(saturation density of nuclei (N_s_)) vs. 1/T. N_s, s_ and N_s, h_ indicate the N_s_ of the spontaneous growth regime and that of the high temperature growth regime, respectively. Their intersection point is marked as T_c_.

**The initial nucleation rate at all temperatures**

From Equation (S24), the initial nucleation rate J can be predicted by the condition dN_x_/dt at t = 0, N_x_ = 0.

$J=(\frac{CR^{2}p_{d}}{N_{0}p_{des}^{2}})(\frac{p_{att,s}}{p_{att}^{2}})$ (S41)

$J=(\frac{C}{N_{0}\upsilon})(\frac{p_{CH_{4}}}{\sqrt{2m_{CH_{4}}kT}}s_{0})^{2}\exp(\frac{2E_{des}+2E_{att}-E_{att,s}-2E_{ad}-E_{d}}{kT})$ (S42)

**Further considerations about temperature dependant growth regimes**

Our growth mechanism was derived by the balance rate equations (Equations S16~S19). From these equations, highly non-linear differential equation (Equation S20) was obtained. To obtain the solution, the ratio, η, was used as a determinant for the temperature dependant growth regime (Equation S21). From beginning to end, all equations were derived by various activation barriers related with the behaviors of C-precursor and C adatom. For better predictions, the properties of the substrate including the dissolution of C adaom into the substrate, the melting and the boiling temperatures of the substrate, the crystalline transformation temperature, and so on, should be considered. For ultrathin γ-Al_2_O_3_/SiO_2_ film on Si case, the highest temperature limit would be ~1200 °C related with the transformation of the crystalline phases^3^. However, after transition temperature of 950 °C as demonstrated at Figure S4, the growth is dominantly governed by the spontaneous dehydrogenation. At this regime, the solution lies in that the number of adsorption sites of CH_4_. Even with the crystalline transformation, the change of the number of sites would be small. After the growth temperature of 1200 °C, the transformed substrate start to have the C solubility at the temperatures > 1427 °C^4^. From this temperature, the predicted growth behaviour would have large differences. Since this temperature, our adsorption based growth model is invalid due to the C solubility.

**Evolution of the overall growth barrier compared to experimental results**

**The catalytic growth regime**

Graphene growth below 900 °C has a nucleation activation energy of E_c_ in the catalytic growth regime, which is expressed as follows.

$E_{c}=-\frac{E_{d}+E_{att}+E_{m}-E_{att,s}-E_{ad}}{3}$ (S43)

Our AFM analysis indicates that the coverage of graphene was less than 1 on γ-Al_2_O_3_ during the growth duration. Under such conditions, it is very difficult to anticipate that E_m_ acts as a barrier. Therefore, E_c_ can be reduced to

$E_{c}=-\frac{E_{d}+E_{att}-E_{att,s}-E_{ad}}{3}$ (S44)

We calculated E_c_ as 0.22 eV, which fitted well with our experimental results. Hence, the derived equations are valid for graphene growth on γ-Al_2_O_3_ substrates.

This equation implies that graphene growth is regulated by the dissociative adsorptions of CH_4_. The generated C adatoms readily diffuse (E_d_ ~0 eV) at the specific sites and make small nuclei without an attachment activation barrier (E_att,s_ ~0 eV) and enlarge with a significant attachment barrier (E_att_ ~0.21 eV). Hence, the formation of small nuclei dominates in comparison to the enlargement of small nuclei, is known to encourage the homogeneous growth of graphene nuclei.

**Evolution of E_d_**

**
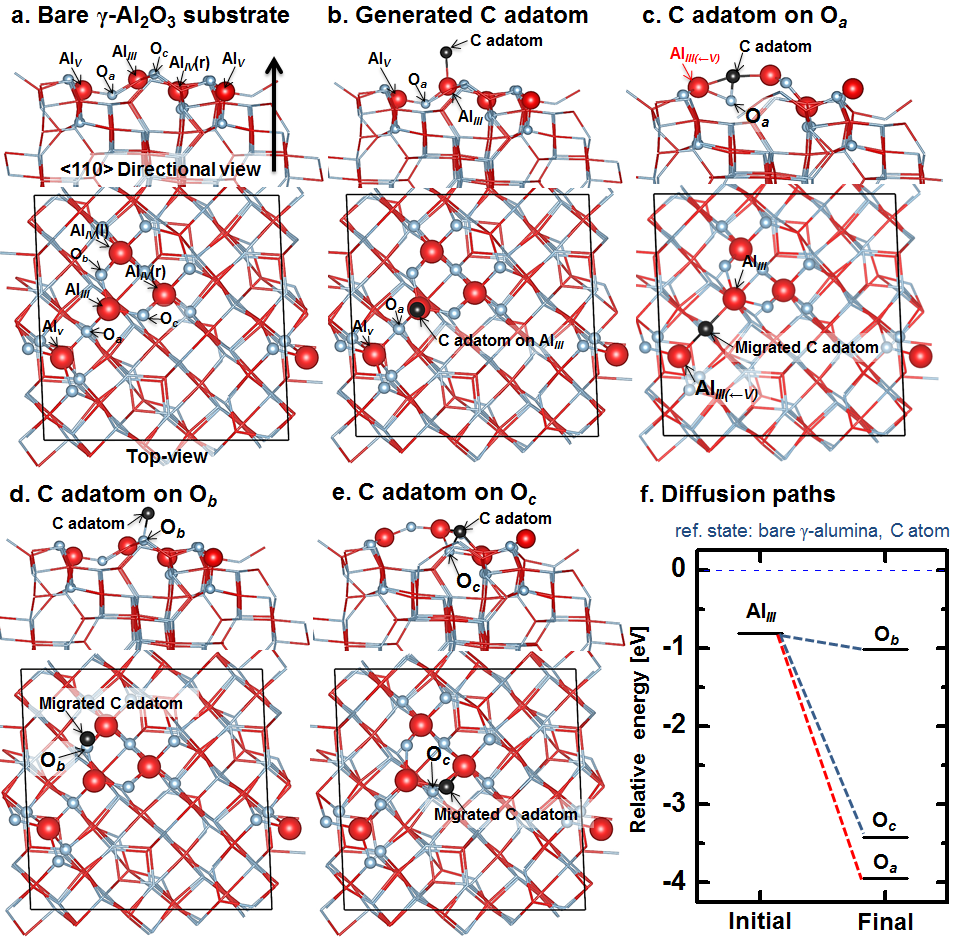
**

**Figure S5.** <110> directional view (top) and top view (bottom) of the **a**, bare γ-Al_2_O_3_ substrate, **b**, generated C adatom obtained from the dissociative adsorption of CH_4_ on Al*_III_*, and the diffusion of the C adatom on specific sites i.e. **c**, O*_a_*, **d**, O*_b_*, and **e**, O*_c_*. O*_a_*, O*_b_*, and O*_c_* are the adjacent Al*_III_*, O sites.**f**, Energy paths for the diffusion of C adatoms from the initial Al*_III_* site to the final specific sites.


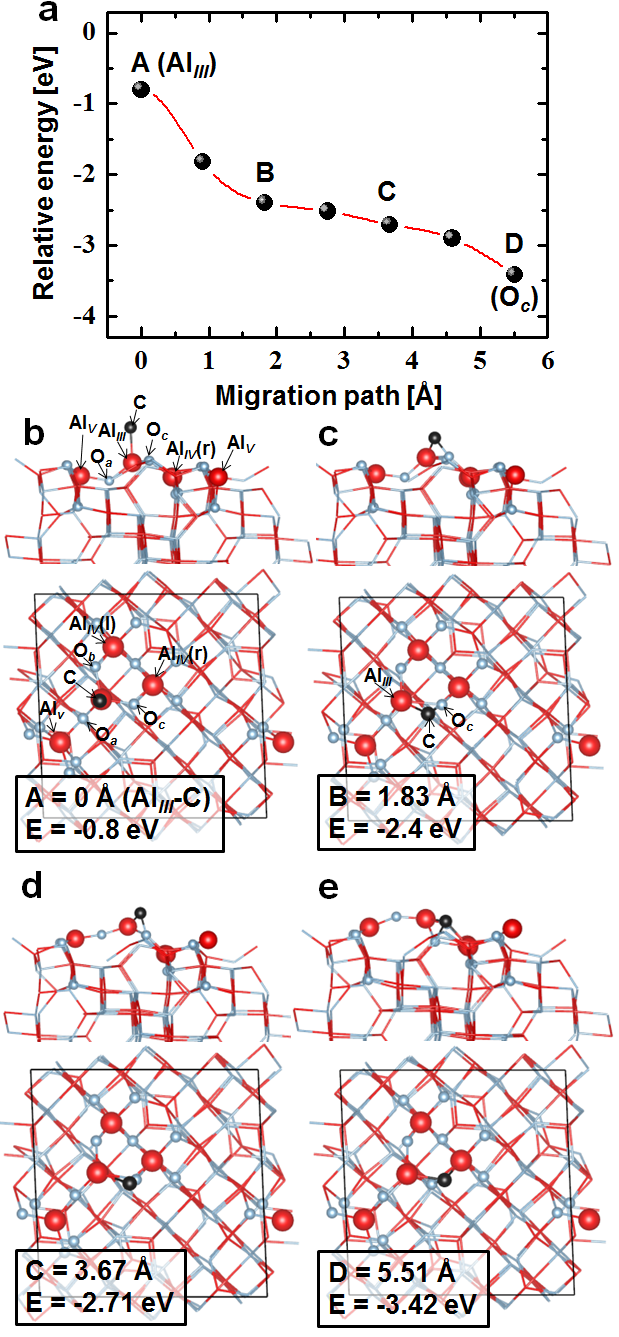


**Figure S6**. **a**, Minimum energy path (MEP) of the generated C adatom from (b) the initial Al*_III_* site to the (e) final O*_a_* site. The diffusion distance of the C adatom of **b**, 0 Å, **c**, 1.8 Å, **d**, 3.7 Å, and **e**, 5.5 Å.

**Formation of C dimer on the O*_a_* site**

We calculated the formation of the most stable dimer by atom-by-atom mode. The result was attached in Figure S7. The most stable C-C bond length on the γ-Al_2_O_3_(110) substrate was 1.28 Å, which is very close to the alkene bond length (sp^2^) of 1.34 Å and is shorter than that of the C-C diatom on flat Ir(111), Ru(0001), and Cu(111) surfaces, which are observed to be 1.40 Å, 1.38 Å, and 1.30 Å, respectively^5^. Hence, the bond length of the C-C dimer on γ-Al_2_O_3_ is a very strong double bond, which would induce C-Al bond weakening, largely. These results support that the formation of nuclei begins from the dimer-preferred feature and is beneficial to the detachability of the graphene grown at an atomic scale^6^. This short atomic length indicates that our graphene sheet has abundant charge density in the bonds, which contributes to the opening of the band gap (E_g_).

**
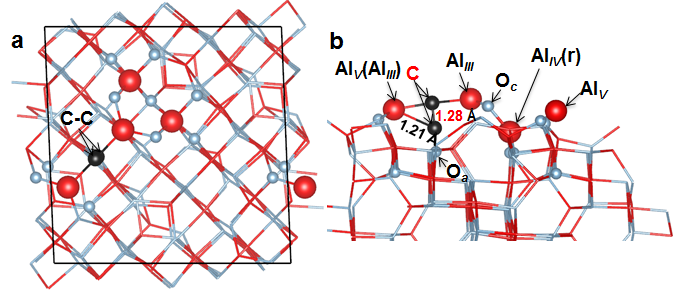
**

**Figure S7. a**, Top view and **b**, side view of the most stable C diatoms identified by the minimum energy path (MEP). The bond lengths of the additional C adatom with Al*_V_* and the previously attached C adatom are1.21 Å and 1.28 Å, respectively.

**AFM analysis of the synthesised graphene**


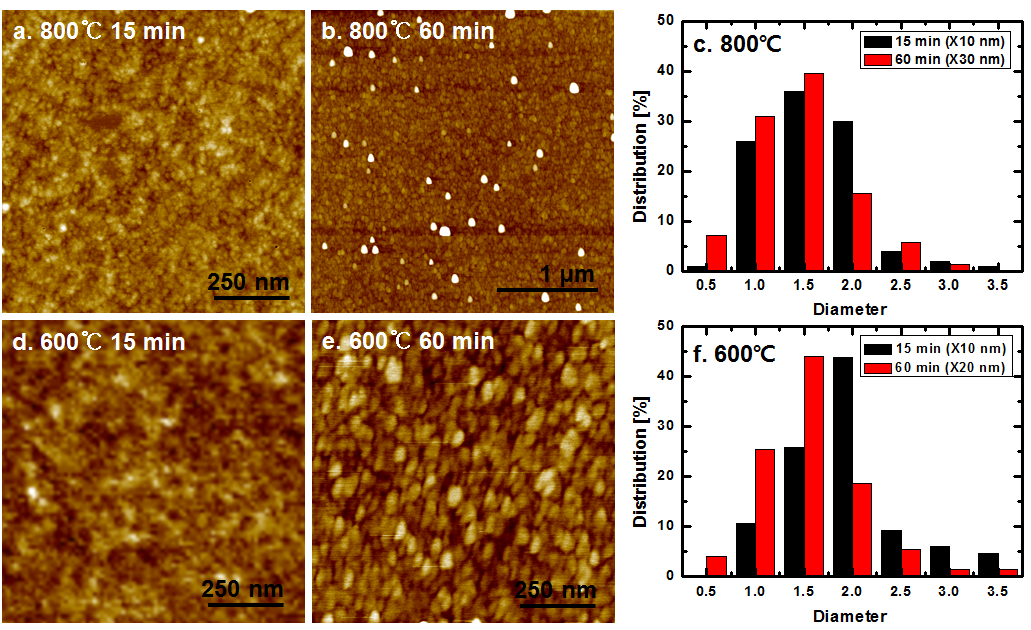


**Figure S8.** AFM images and the areal distributions of the graphene grains grown on the γ-Al_2_O_3_ substrates for different growth temperatures and times. **a**, 800 °C, 15 min, **b**, 800 °C, 60 min, **d**, 600 °C, 15 min, and **e**, 600 °C, 60 min. **c**, and **f**, are their areal distributions grown at 800 °C and 600 °C, respectively.

**Table S1.** Analysis of the AFM images of the graphene grains grown on the γ-Al_2_O_3_ substrate at different growth temperatures and times.

| **Growth time** |  | **600** °C | **800** °C | **1050** °C |
| --- | --- | --- | --- | --- |
| **15 min** | Diameter [nm] | 14.6 | 16.0 | 26.4 |
|  | Area [nm^2^] | 167 | 201 | 550 |
|  | Coverage [%] | ~70 | 94 | ~100 |
|  | number of grains/μm^2^ | 4200 | 4677 | 1819 |
| **20 min** | Diameter [nm] |  |  | 52 |
|  | Area [nm^2^] |  |  | 2127 |
|  | Coverage [%] |  |  | 100 |
|  | number of grains/μm^2^ |  |  | 470 |
| **60 min** | Diameter [nm] | 30.5 | 43.0 |  |
|  | Area [nm^2^] | 730 | 1451 |  |
|  | Coverage [%] | 83.4 | ~99 |  |
|  | number of grains/μm^2^ | 1142 | 682 |  |


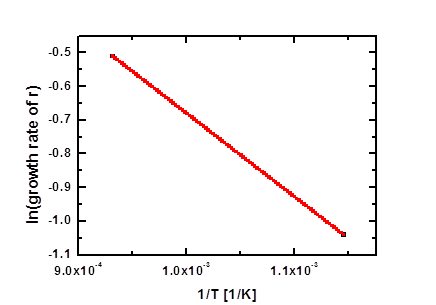


**Figure S9.** Arrhenius plot of the graphene growth rate obtained by plotting *ln*(growth rate of r, $\sqrt{A_{G}}$) and 1/T, which were acquired from the AFM analysis.

**Evolution of E_att_ and E_form_**

E_att_ is closely related with the enlargement of nuclei. A simple model considering the growth rate of graphene islands by attachment and detachment of C adatoms can be defined by edge controlled kinetics, as shown in Equation (8) ^2^.

$\frac{dA_{G}}{dt}=k_{1}c\sqrt{A_{G}}-k_{2}\sqrt{A_{G}}$ (S45)

In the above equation, A_G_ is the area of the graphene island, k_1_ and k_2_ are the attaching and the detaching rate constants of the C adatom at the perimeters of the graphene islands ($\sqrt{A_{G}}$), and c is the concentration of the free C adatoms generated by the dissociative adsorption of CH_4_. The rate constants k_1_ and k_2_ are exponential functions of E_att_ and E_det_, respectively.

Our calculations indicate that the E_det_ of C atom from graphene is 9.24 eV. Therefore, k_2_ can be considered as negligible under the presently employed CVD conditions.

$\frac{dA_{G}}{dt}=k_{1}c\sqrt{A_{G}}$ (S46)

We analysed the graphene growth rates of 315.4 nm^2^ min^-1^, 28 nm^2^ min^-1^, and 12 nm^2^ min^-1^ at 1050 °C, 800 °C, and 600 °C, respectively, by varying the growth time (see Figures S8, S9, and Table S1. By plotting the Arrhenius diagram of *ln*(growth rate of $\sqrt{A_{G}}$) against 1/T, we obtained E_att_ as 0.21 eV from Figure S9. We also obtained the formation energy, E_form_, as 0.31 eV and 0.32 eV from the DFT calculations (for a free-standing graphene, the value is 0.27 eV and the attraction between graphene and γ-Al_2_O_3_ contributes 0.04 eV/C atom) and from the experimental results by AFM analysis, respectively.

These values look quite small compared to the activation energies of 1-2 eV for carbon growth on metal surfaces^2,7,8^. However, these values (1-2 eV) imply that the growths are limited by C diffusion and C solubility in the bulk metal catalyst or substrates^7^. In the case of Ni, the areal growth rate of a carbon nanotube possesses an activation energy of 2.02 eV energy, which consists of a solubility barrier of the C adatom (E_sol_) of 0.42 eV and the bulk diffusion barrier of the C atom into Ni film (which amounts to 1.62 eV)^8^. This type of growth is based on the catalytic liquid of the vapour-liquid-solid method yielding a vapour to supersaturation, leading to segregation or precipitation during growth. Clearly, in the above described mechanism, growth from the surface diffusion was neglected because the sum of the bulk diffusion barrier and E_sol_ is nearly equal to the overall activation barrier.

If the graphene growth occurs only by surface diffusion on the transition metal, E_form_ of the end of a C chain is reduced from ~3.5 eV to ~0.20 eV on the Ni (111) surface^9^. Such end formations and ring formations are strictly related with the enlargement of the graphene nuclei. Therefore, the E_att_ of 0.21 eV and E_form_ of 0.31 eV we obtained may indicate that the catalytic activity of γ-Al_2_O_3_ is comparable to that of Ni. This implies that graphene formation on γ-Al_2_O_3_ can be achieved at a temperature comparable to nearly the room temperature synthesis of graphene on Ni^10^.

**Further interpretation of growth related factors**

Our nucleation and growth equations represented in Equations (4), (6), and (7) show an Arrhenius form, which consists of prefactors and temperature dependent exponents of E_a_. The prefactors mainly reflect the process parameters, including the partial pressure and molecular weight of C precursor and the growth temperature. The exponent of E_a_ describes the probability. Our equations show a weak temperature dependence of the prefactors and a very strong temperature dependence of the exponent. By assuming the same process parameters, the growth phenomena related with material characteristics can be interpreted.

C solubility, E_sol_, and E_d_ determine the growth mechanism. In the case of graphene growth, which follows surface adsorption, C solubility should be negligible or E_sol_ should be significantly larger than E_d_. Paradoxically, Ni has comparable E_sol_ and E_d_. Hence, the probabilities of C adatom diffusing on the surface and dissolving into the bulk are identical at a point. C adatom diffusion and the dissolution probability of C adatom into the bulk Ni follow a cumulative distribution function. Therefore, the formation of graphene on Ni basically follows the segregation or precipitation mechanism.

J and N_s_ indicate the density of the graphene nuclei during the initial and final stages of growth, respectively. Comparing the J and N_s_ of γ-Al_2_O_3_ to Cu, the ratios of the values for γ-Al_2_O_3_ to that of Cu exhibit distinct differences in terms of the material characteristics. For a growth temperature of 800 °C, the J and N_s_ ratios of γ-Al_2_O_3_/Cu are 3.49 × 10^39^ (which was evaluated by assuming E_des_ as 6.06 eV for comparing γ-Al_2_O_3_ and Cu) and 1.69 × 10^5^, respectively. Theoretically, graphene nuclei at initial growth stages cannot be formed on the Cu surface because of its large E_ad_ of 3.4-4.1 eV. This corresponds to the results of the thermodynamic simulation of graphene growth on Cu surface, which proved that the existence of C atoms are thermodynamically unfavorable^11^. When the growth time is 15 min at 800 °C, the average diameter of the graphene nuclei can be predicted as 16 nm on γ-Al_2_O_3_ and as 2.1 μm on Cu under identical CVD conditions. Therefore, we can conclude that γ-Al_2_O_3_ and Cu are good for growing nanometre and micrometre sized grains, respectively. On the contrary, Cu cannot form the nanometre grains in a close-packed form. Customization of grain sizes ranging from nanograins to micrograins can be easily achieved with γ-Al_2_O_3_, which would open wide opportunities toward potential applications on account of the relatively easy control of band gap opening as well as grain boundary effects.

**Raman analysis**

Figure S10 shows the Raman spectra of the graphene samples grown at 600, 800, and 1050 °C, respectively.

**
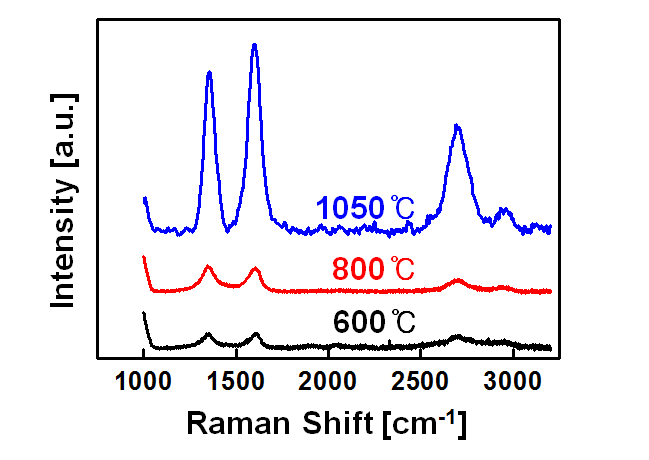
**

**Figure S10.** Raman spectra of samples Figure 4(a), (b) and (c).

**Table S2.** The specific analysis of the Raman spectra as a function of the growth temperature.

| Growth  Temperature  [°C] | D peak  (FWHM)  [cm^-1^] | G peak  (FWHM)  [cm^-1^] | 2D peak  (FWHM)  [cm^-1^] | I_G_/I_2D_ | I_G_/I_Si_ |
| --- | --- | --- | --- | --- | --- |
| 600 | 1349  (70) | 1601  (58) | 2709  (180) | 1.69 | 0.39 |
| 800 | 1349  (70) | 1602  (58) | 2699  (115) | 1.94 | 0.62 |
| 1050 | 1357  (55) | 1600  (67) | 2701  (99) | 1.89 | 5.4 |

**Growth optimization**

It is essential to optimize any CVD synthesis. At first, we fixed the growth temperature and varied the flow ratios of CH_4_ and H_2_ and their total amounts to obtain the best quality of synthesized graphene. Raman analysis and measurements of sheet resistances (R_s_) were performed to evaluate the quality of the grown graphene samples. As shown in Figure S11a, the overall structures of the grown graphene is bilayered, as understood from the intensity ratios of *I_2D_*/*I_G_*. The grades of the defectiveness of the grown graphenes were evaluated by *I_D_*/*I_G_* intensity ratios. From this analysis, the optimized condition was found to involve 25-50 sccm of H_2_ at a CH_4_ flow of 850 sccm. H_2_–free conditions were also acceptable in view of the small changes in the I_D_/I_G_ relative to the flows of H_2_ at 25-50 sccm. Hence, we can grow high-quality graphene under H_2_-free conditions. This feature can be a strong point of the synthesis of graphene in comparison to synthesis on transition metals including, Ni and Cu. To obtain the high-quality graphene on transition metals, the growth environments involve flows of H_2_, which are larger than the flows of CH_4_. Despite the numerous reports on the role H_2_, the concept remains unclear. In of the view of chemical potential, the chemical potential of C is determined by the relationship between the equilibrium of CH_4_ and H_2_^12^. When proving the growth mechanism, the chemical potential is directly related to the equilibrium and the nucleation concentration of the C adatom, which are key factors determining the formation energy, the nucleation, and the growth of the graphene^2,7^.

From the ratio of *I_Si_*/*I_G_* in the Figure S11b, the domain sizes of the graphene samples were found to be almost identical up to H_2_ flows of 400 sccm. However, the measured R_s_ exhibited almost a linear relationship when the H_2_ flow was increased until 200 sccm, as shown in Figure 4f and then abruptly increased, as shown in Figure S11c. The homogeneity based on the standard deviations of R_s_ was almost constant (< ~1% standard deviation) for samples synthesised with flows up to 200 sccm and then, the values abruptly increased for samples grown with flow rates beyond 200 sccm.

**
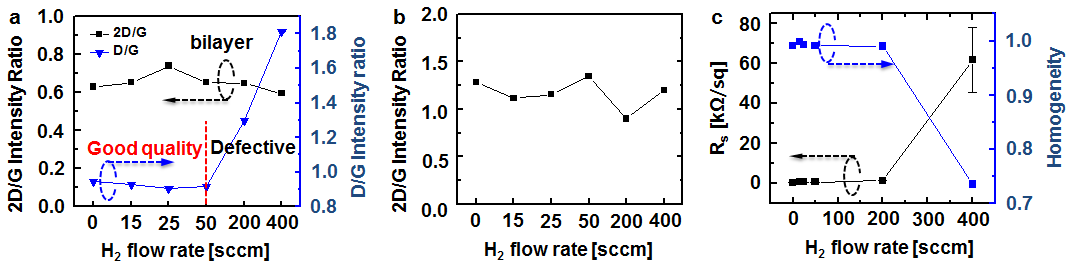
**

**Figure S11. Graphene samples were grown at a fixed CH_4_ flow rate of 850 sccm by varying the H_2_ flow rate from 0 to 400 sccm.** **a**, I_2D_/I_G_ ratios, I_D_/I_G_ ratios, and **b**, I_Si_/I_G_ ratios obtained from the Raman analysis. **c**, R_s_ and the uniformity of R_s_ of the samples. Homogeneity was estimated by (R_s_-R_s,stdev_)/R_s_, where R_s, stdev_ is standard deviation of R_s_.

**XPS analysis**

X-ray photoelectron spectroscopy (XPS) was used to estimate the quality of the grown graphene between 600 °C and 1050 °C and the conditions of the γ-Al_2_O_3_ substrate. Four kinds of high-resolution spectra were acquired for the analysis of C 1s (Figure S12), N 1s, O 1s, and Al 2p (Figure S13a, b and c, respectively.).

As the Al*_III_* sites show a higher reactivity toward N_2_ than CH_4_^13,14^, N-doping may occur if any air permeation exists during the CVD process. Therefore, it is important to check the N 1s spectrum to ensure the air tightness of the CVD system. As shown in Figure S13a, there was no characteristic peak in the range of N 1s. Hence, our CVD system is well made for the synthesis of graphene and the grown graphene does not contain N.

Figure S13b shows the high-resolution spectrum of O 1s. As γ-Al_2_O_3_ is metastable, the compositional and structural properties are dependent on the preparation conditions, yielding the shift of binding energies (BE) from 531.08 eV to 531.5 eV (BE of α-Al_2_O_3_ = 530.68 eV^15^, BE of amorphous Al_2_O_3_ = 531.4 eV^16^). From the peak analysis, the major peak is at 531.38 eV (88.0%) with a sub peak at 531.08 eV (1.0%), which is related to pure γ-Al_2_O_3_. There were two C related peaks at 534.3 eV (3.7%) for C-O and at 532.8 eV (7.4%) for C=O.

Figure S13c shows the high-resolution spectrum of Al 2p. This peak is also dependent on the preparation conditions. The BE difference of O 1s and Al 2p can be indicative of fully oxidized O-Al-O bonds of 456.6 eV^6,17^. A BE difference of ~1% corresponds to the BE of 456.6 eV, and 88% corresponds to 456.2 eV, which is indicative of not fully oxidized O-Al-O bonding. Hence, our Al_2_O_3_ substrate is composed of a metastable phase. Characteristic shapes of the Al 2p peaks are also used to classify the crystalline polymorphs of Al_2_O_3_^6^. According to DFT calculations^6^, the spectrum of γ-Al_2_O_3_ consists of one peak (Al*_IV_* or Al*_V_*) with a small shoulder (Al*_III_*), showing that the BE differences are excessively small to be well resolved, as shown in Figure S13c. This shape is unique for γ-Al_2_O_3_. Therefore, we conclude that the small shoulder (peak) at 73.64 eV belongs to Al*_III_* and the main peak at 74.58 eV belongs to Al*_IV_* or Al*_V_*. Moreover, the areal compositions of these peaks is 1:3, which supports that these peaks originated from the (110) surface of γ-Al_2_O_3_. Consequently, we can conclude that the (110) surface is predominant in our γ-Al_2_O_3_ substrate. The other two peaks at 75.44 eV and 76.02 eV were induced by the stabilization of the adsorption of graphene with Al*_III_* and Al*_IV_*, respectively. The BE differences are 1.8 eV and 1.5 eV, indicating that the tendency of Al*_III_* sites to undergo stabilization is higher. Moreover, the compositional ratios of 1: 1 also support this feature related with the instability or the catalytic activity of the Al*_III_* sites. Additionally, this result can be seen as an evidence for surface reconstruction by the physical coverage of the grown graphene. Figure 4e shows the changes in the relative contents as a function of the growth temperatures. At higher growth temperatures, the sp^3^ portion increased and the portion associated with oxygen decreased. The overall quality of sp^2^ content was almost fixed at ~90%, and the total carbon content in the graphene sheet was > 97%, which is comparable or superior to that of pristine graphite of ~96%^18^.


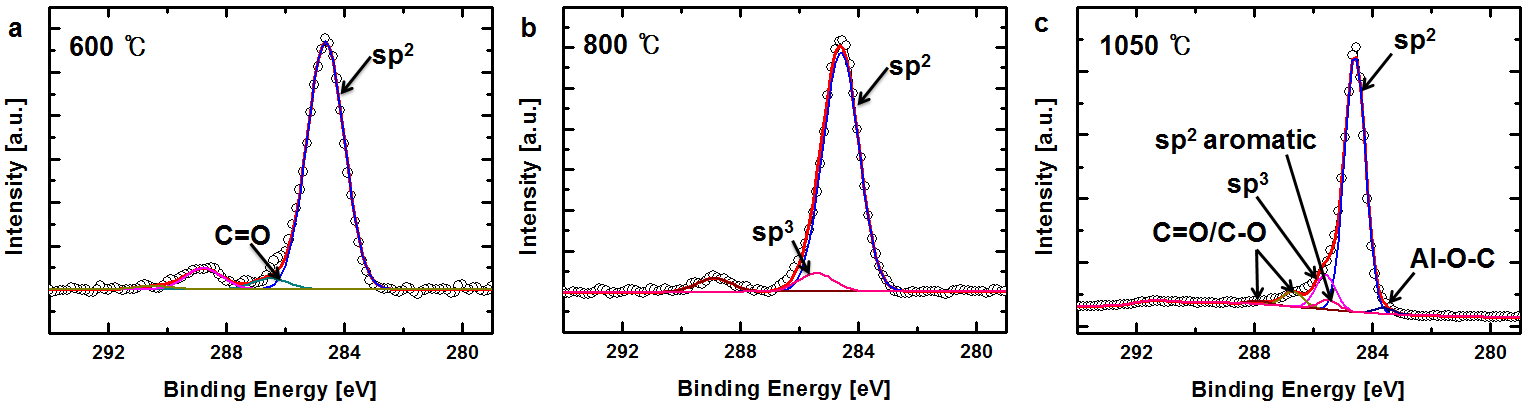


**Figure S12.** High-resolution C1sXPS profiles of graphene grown at **a**, 600 °C, **b**, 800 °C, and **c**, 1050 °C, respectively.


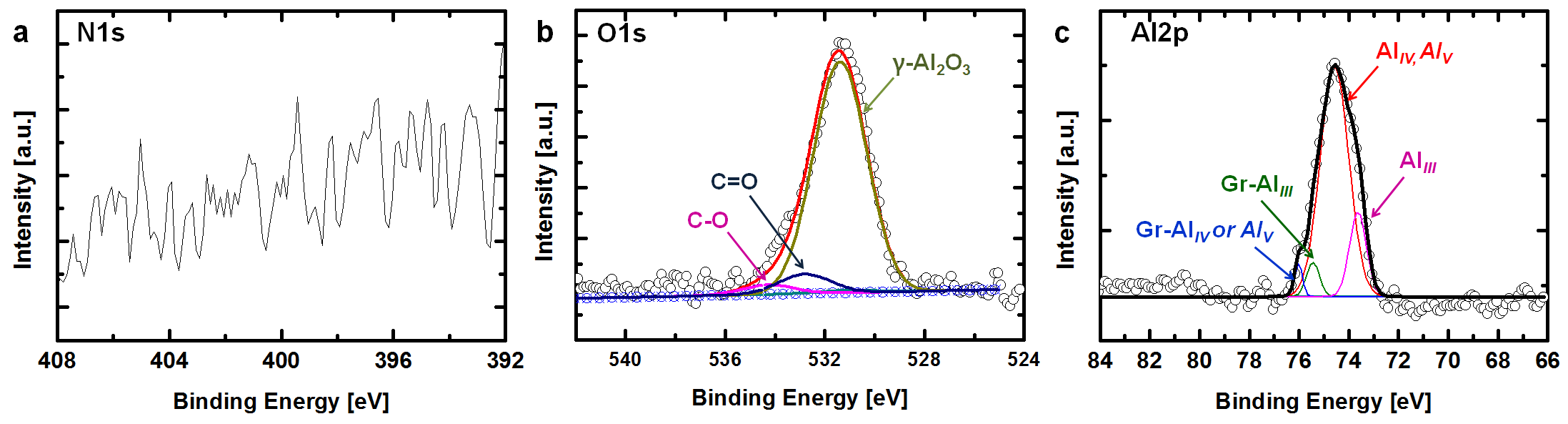


**Figure S13.** High-resolution XPS profiles of **a**, N 1s, **b**, Al 2p, and **c**, O1s.

**XRD analysis**

**Evolution of substrate crystalline phase**

By comparing our X-ray diffraction (XRD) data with the crystallographic online database and the international centre for diffraction data database, the crystalline phase of the γ-Al_2_O_3_ substrate formed during the first growth of graphene remained unaltered during growth and re-growth. The XRD data (Figure S14a) showed the (110), (220), (400), and (440) peaks. In addition, the XRD data indicated the presence of weak (400) and strong (440) peaks, which demonstrate that the structure of the substrate is a defect spinel and that the (110) surface is predominant. Additionally, the (222) and (444) peaks, corresponding to the (111) surface, were absent. Such results are indicative of typical crystalline features of γ-Al_2_O_3_. Moreover, the distinctive characteristic peaks of ξ, φ, ψ, κ, θ, and α phases were absent. These results support that the growth conditions used for the CVD including the re-growth did not transform the crystalline phase of Al_2_O_3_. Additionally, these results guarantee the reproducibility of the graphene synthesis after reusing the Al_2_O_3_ substrate.

**XRD analysis for the evaluation of graphene quality**

We also analysed our graphene samples by using XRD to confirm the quality of graphene as shown at Figure S14b and c. The peak at 2θ ranging from 10° to 35° is indicative of the quality of the grown graphene. The graphene sample grown at 1050 °C for 1 h shows a broad peak at 2θ = 25.9° corresponding to a d-spacing of 3.44 Å, which is indicative of a lower degree of crystallization and the presence of some defects^19,20^. Three sharp peaks were present at 2θ = 26.5°, 26.3°, and 26.1° corresponding to the d-spacing of 3.36 Å, 3.38 Å, and 3.41 Å, respectively. In a reported synthesis of few-layered graphene, an interlayer spacing of 3.39 Å from a sharp peak at 2θ = 26.3° was found to correspond with the highly crystalline structure^20^. Since bilayered graphene on Al_2_O_3_ substrate has weaker adsorption energy, the interlayer spacing would be smaller than that observed in the case of that on the SiO_2_. Therefore, we think that the interlayer distance of 3.36 Å from the peak at 2θ = 26.5° might correspond with the highly crystalline structure of graphene on the γ-Al_2_O_3_ substrate. Comparatively, the distance of 3.38 nm and 3.41 nm from the peaks at 2θ = 26.3° and 26.1° might originate from the complicated nature of disorder in the graphene sheets^20^ and the strain stored during the areal expansion of grains or the surface of γ-Al_2_O_3_. Comparing the XRD data of samples prepared at 600 °C, 800 °C, and 1050 °C, the quality of the graphene grown at 600 °C and 800 °C is better than that grown at 1050 °C because in the corresponding XRD profiles, the peaks at 25.7°, 26.1°, and 26.3° are absent. In addition, our samples also do not show the peak at 2θ ≈ 11.3° (7.77 Å), corresponding to graphene oxides. From the XRD profiles, we can conclude that high quality graphene has been synthesized and the catalytically grown graphene has better quality than the spontaneously grown one.

**Theoretical discussion about interlayer distance of bilayered graphene**

To analyse these characteristic peaks in Figure S14b and c, we referred to the interlayer distances of graphite and bilayer graphene obtained by simulations^11,19,21-23^ and experimental results^20-22^. The interlayer distance of AB-stacked and AA-stacked hexagonal graphites are estimated to range from 3.31 Å (Experimental: 3.33)^21^, ~3.35 Å (Experimental: 3.35 Å)^22^, to 3.60 Å by local density approximation (LDA). We excluded the results from van der Waals DFT (VDWDFT), which tends to overestimate the equilibrium distance up to 3.59 and 3.65 Å^22,23^. The interlayer distance of AB-stacked and AA-stacked bilayer graphenes are estimated to be 3.35 Å (3.58 Å by generalized gradient approximation (GGA))^19^ and as 3.60 Å by LDA^22^. However, considering the edge chirality and size, the distance can be reduced to up to ~3.1 Å because of changes in the charge density^9^. This factor also participates in the opening of the graphene band gap. However, these kinds of simulations fail to reflect substrate effects, including the fixed charges. Consequently the experimental results from bilayered graphene are sufficient to predict the interlayer distance.


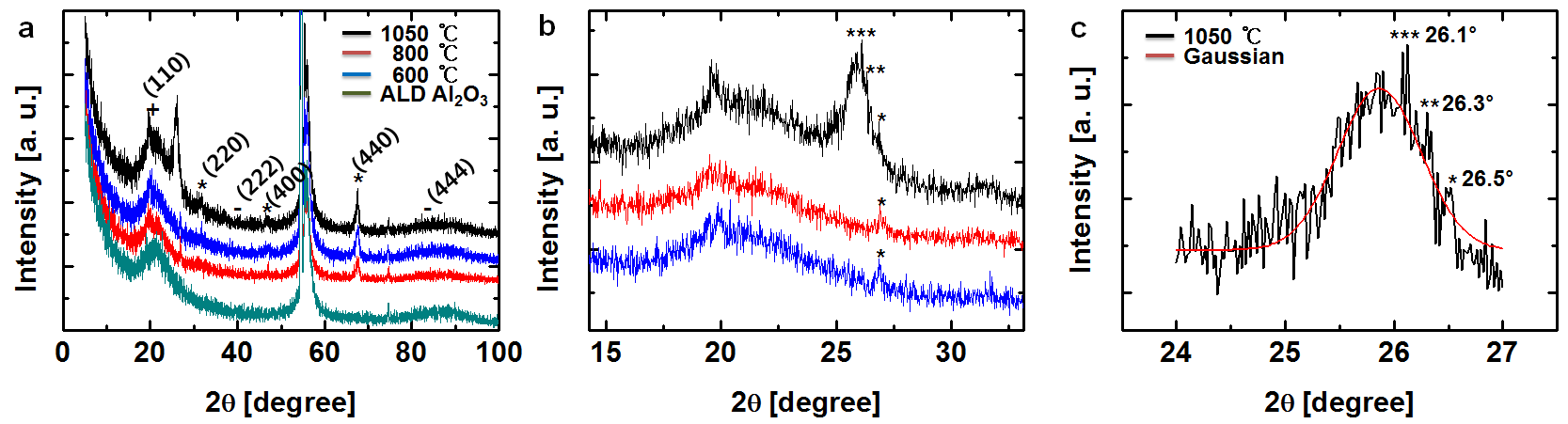


**Figure S14. a**, Wide and **b**, middle range XRD patterns of graphene samples grown at 1050 °C, 800 °C, and 600 °C for 1 h with 850 sccm of CH_4_ and 50 sccm of H_2_. **c**, Narrow range XRD patterns of graphene sample grown at 1050 °C. The red line is the Gaussian plot for evaluating the centre of the peak, which corresponds to a spacing of 3.44 Å, indicative of a lower degree of crystallization and the presence of some defects. The lowest pattern of **a** belongs to the ALD Al_2_O_3_, which is the reference pattern. Peaks of **a** marked by + and - indicate the visible and invisible characteristic peaks of γ-Al_2_O_3_. Peaks of **b** and **c** marked by *, **, *** indicate peaks at θ = 26.5°, 26.3°, and 26.1°, respectively, corresponding to the graphene d-spacings of 3.36 Å, 3.38 Å, and 3.41 Å, respectively.

**Optical analysis**

**Universal transmittance of freestanding graphene**

The universal transmittance of freestanding graphene, T_free_, can be derived by using Frensnel equations for a thin film with a universal conductance of σ^2d^ = e^2^/4ħ as shown in Equation (47)^24,25^.

$T_{free}=(1+\pi\alpha/2)^{-2}\approx1-\pi\alpha\approx0.977$ (S47)

In the above equation, α is the fine structure constant^24,25^.

From this derivation, single layer graphene, which is free spaced (with refractive index, n = 1) can absorb ~2.3% of the incident light independent of its wavelength.

**Graphene mode-locked laser setup**

Figure S15 represents the experimental setup of fibre ring laser in order to demonstrate the ultrafast pulses formed by our ‘graphene sticker’.

**
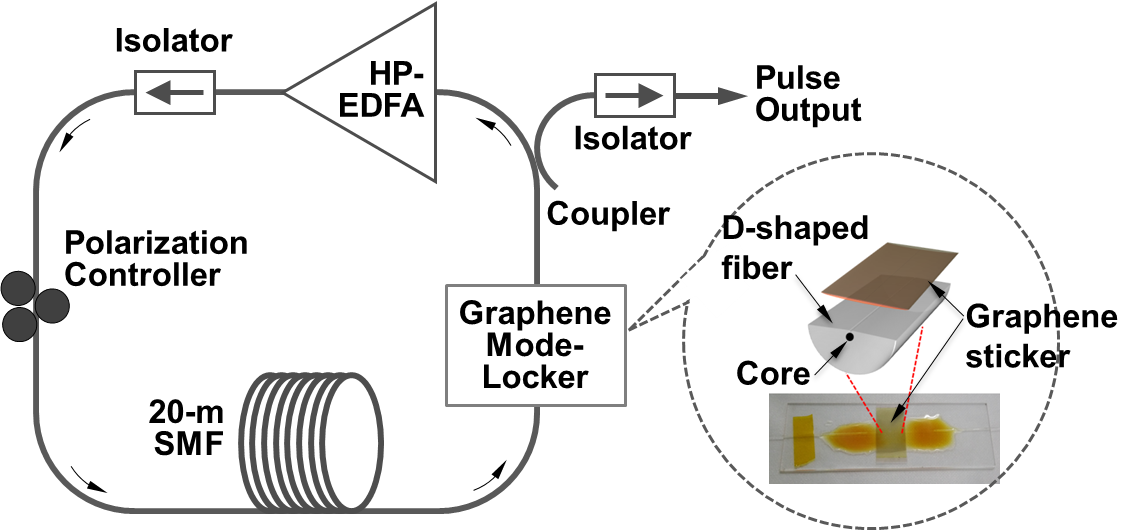
**

**Figure S15**. Ultrafast mode-locked laser setup. Graphene sticker as a nonlinear saturable absorber was attached on D-shaped fibre to form ultrafast laser pulses in a fibre ring laser cavity. (HP-EDFA: High Power Erbium-Doped Fibre Amplifier, SMF: Single-Mode Fibre)

**SI references**

1. Robinson, V. N. & Robins, J. L. Nucleation kinetics of gold deposited onto UHV cleaved surfaces of NaCl and KBr. *Thin Solid Films* **20**, 155-175 (1974).

2. Kim, H. *et al.* Activation energy paths for graphene nucleation and growth on Cu. *ACS Nano* **6**, 3614-3623 (2012).

3. Tahir, D. *et al*. Electronic and optical properties of Al_2_O_3_/SiO_2_ thin films grown on Si substrate. *J. Phys. D: Appl. Phys.* **43**, 255301 (2010).

4. Menchavez, R. L., Fuji, M., Yamakawa, T., Endo, T. & Takahashi. M. Investigation of phase composition in dense and porous gelcast alumina sintered under argon atmosphere. *Mater. Sci. Forum* **561-565**, 2123 (2007).

5. Chen, H., Zhu, W. G. & Zhang, Z. Y. Contrasting behavior of carbon nucleation in the initial stages of graphene epitaxial growth on stepped metal surfaces. *Phys. Rev. Lett.* **104**, 186101 (2010).

6. Lizarraga, R., Holmstrom, E., Parker, S. C. & Arrouvel, C. Structural characterization of amorphous alumina and its polymorphs from first-principles XPS and NMR calculations. *Phys. Rev. B* **83**, 094201 (2011).

7. Loginova, E., Bartelt, N. C., Feibelman, P. J. & McCarty, K. F. Evidence for graphene growth by C cluster attachment. *New J. Phys.* **10**, 093026 (2008).

8. Kaatz, F. H., Siegal, M. P., Overmyer, D. L., Provencio, P. P. & Tallant, D. R. Thermodynamic model for growth mechanisms of multiwall carbon nanotubes. *Appl. Phys. Lett.* **89**, 241915 (2006).

9. Gao, J. F., Yip, J., Zhao, J. J., Yakobson, B. I. & Ding, F. Graphene nucleation on transition metal surface: structure transformation and role of the metal step edge. *J. Am. Chem. Soc.* **133**, 5009-5015 (2011).

10. Kwak, J. *et al.* Near room-temperature synthesis of transfer-free graphene films. *Nat Commun.* **3**, 645 (2012).

11. Lam, K. T. & Liang, G. C. An ab initio study on energy gap of bilayer graphene nanoribbons with armchair edges. *Appl. Phys. Lett.* **92**, 223106 (2008).

12. Zhang, W. H., Wu, P., Li, Z. Y. & Yang, J. L. First-principles thermodynamics of graphene growth on Cu surfaces. *J. Phys. Chem. C* **115**, 17782-17787 (2011).

13. Wischert, R., Laurent, P., Coperet, C., Delbecq, F. & Sautet, P. gamma-Alumina: the essential and unexpected role of water for the structure, stability, and reactivity of "defect" sites. *J. Am. Chem. Soc.* **134**, 14430-14449 (2012).

14. Wischert, R., Coperet, C., Delbecq, F. & Sautet, P. Dinitrogen: a selective probe for tri-coordinate Al "defect" sites on alumina. *Chem. Commun.* **47**, 4890-4892 (2011).

15. Rotole, J. A. & Sherwood, P. M. A. Valence band X-ray photoelectron spectroscopic studies to distinguish between oxidized aluminum species. *J. Vac. Sci. Technol. A* **17**, 1091-1096 (1999).

16. Lee, B. K. *et al.* Conformal Al_2_O_3_ dielectric layer deposited by atomic layer deposition for graphene-based nanoelectronics. *Appl. Phys. Lett.* **92**, 203102 (2008).

17. Renault, O., Gosset, L. G., Rouchon, D. & Ermolieff, A. Angle-resolved X-ray photoelectron spectroscopy of ultrathin Al_2_O_3_ films grown by atomic layer deposition. *J. Vac. Sci. Technol. A-Vacuum Surfaces and Films* **20**, 1867-1876 (2002).

18. Yi, M., Shen, Z. G., Zhang, X. J. & Ma, S. L. Achieving concentrated graphene dispersions in water/acetone mixtures by the strategy of tailoring Hansen solubility parameters. *J. Phys. D Appl. Phys.* **46**, 025301 (2013).

19. Yoshizawa, K., Yumura, T., Yamabe, T. & Bandow, S. The role of orbital interactions in determining the interlayer spacing in graphite slabs. *J. Am. Chem. Soc.* **122**, 11871-11875 (2000).

20. Wu, Y. P. *et al.* Efficient and large-scale synthesis of few-layered graphene using an arc-discharge method and conductivity studies of the resulting films. *Nano Res.* **3**, 661-669 (2010).

21. Taut, M., Koepernik, K. & Richter, M. Electronic structure of stacking faults in hexagonal graphite. *Phys. Rev. B* **88**, 205411 (2013).

22. Alam, M. S., Lin, J. B. & Saito, M. First-principles calculation of the interlayer distance of the two-layer graphene. *Jpn. J. Appl. Phys.* **50**, 080213 (2011).

23. Langreth, D. C. *et al.* A density functional for sparse matter. *J. Phys.-Condens. Mat.* **21**, 084203 (2009).

24. Kuzmenko, A. B., van Heumen, E., Carbone, F. & van der Marel, D. Universal optical conductance of graphite. *Phys. Rev. Lett.* **100**, 117401 (2008).

25. Avouris, P. Graphene: electronic and photonic properties and devices. *Nano Lett.* **10**, 4285-4294 (2010).
